# Research Progress of Electrochemical Machining Technology in Surface Processing: A Review

**DOI:** 10.3390/mi16101174

**Published:** 2025-10-16

**Authors:** Yiran Wang, Yong Yang, Chaoyang Han, Guibing Pang, Shuangjiao Fan, Yunchao Xu, Zhen He, Jianru Fang

**Affiliations:** 1 School of Mechanical Engineering & Automation, Dalian Polytechnic University, Dalian 116000, China; 2 Dalian Institute of Metrology Inspection and Testing Co., Ltd., Dalian 116000, China; 3 Dalian Yaming Auto Parts Co., Ltd., Dalian 116000, China

**Keywords:** electrochemical machining, difficult-to-machine materials, synergistic mechanism, multi-energy field processing, surface treatment

## Abstract

Traditional mechanical processing techniques are confronted with significant challenges when machining advanced materials possessing excellent mechanical properties. Electrochemical machining (ECM), as a material removal technology based on the principle of anodic dissolution, demonstrates distinctive advantages including the absence of contact stress, independence from material hardness, and elimination of mechanical residual stress and recast layers. These characteristics render ECM particularly suitable for high-precision applications requiring superior surface quality. This review systematically summarizes the applications, recent progress, and current challenges of ECM in surface processing. According to diverse surface requirements, ECM technology is classified into two core directions based on primary objectives. The first direction focuses on surface quality enhancement, where nanoscale planarization, residual stress reduction, and uniform surface performance are achieved through precise regulation of anodic dissolution. The second direction concerns material shaping, which is subdivided into macro-scale and micro-scale processing. Macro-scale forming combines electrochemical dissolution with mechanical action to maintain high material removal rate (MRR) while achieving micron-level precision. Micro-scale forming employs nanosecond pulse power supplies and precision electrode/mask designs to overcome manufacturing limitations of micro-nano features on hard-brittle materials. Despite progress achieved, key technical bottlenecks persist, including unstable dynamic control of the inter-electrode gap, environmental concerns regarding electrolytes, and tooling degradation. Future research should prioritize the development of green processing technologies, intelligent control systems, multi-scale manufacturing strategies, and multi-energy field hybrid technologies to enhance the capability of ECM in meeting increasingly stringent surface requirements for advanced materials.

## 1. Introduction

### 1.1. Technical Background and Challenges of Surface Treatment

With the increasingly stringent requirements for service performance of key components, increasing numbers of advanced materials with excellent properties such as high-temperature resistance, high stiffness, and creep resistance are widely used in aerospace, military industry, nuclear power and other fields. However, it is noteworthy that a substantial proportion of these materials possess inherent characteristics such as high hardness, superior strength, and low thermal conductivity, which are recognized as detrimental to machining operations. Consequently, the fundamental challenges regarding effective processing methodologies for these materials, particularly the optimization of surface quality through roughness reduction during machining procedures, have been identified as key research priorities, as documented in recent works [1,2,3].

The surface treatment of these difficult-to-machine materials encounters multiple technical constraints, wherein the compromised process adaptability induced by inherent material characteristics is particularly pronounced. During the processing of titanium alloys, the inherent high chemical reactivity leads to significant tool adhesion and diffusion wear, while the low thermal conductivity intensifies the accumulation of local heat in the cutting zone. Meanwhile, the characteristic of titanium alloys maintaining mechanical strength at high temperatures will accelerate the degradation of tool materials and cause heat-induced dimensional stability problems. The above factors jointly lead to poor adaptability of the processing technology [4]. Due to the inherent material properties of nickel-based alloys, they pose substantial challenges in practical processing. The synergistic interaction of elevated hardness, superior strength, and low thermal conductivity has been demonstrated to induce excessive cutting forces and accelerated tool wear mechanisms. Furthermore, pronounced work hardening tendencies are consistently observed during material removal processes. The localized concentration of cutting heat, resulting from these intrinsic characteristics, has been shown to promote thermal deformation phenomena and surface integrity deterioration. These cumulative influences eventually lead to a decline in processing efficiency and surface quality, thereby greatly limiting the adaptability of the process [5].

Conventional mechanical machining technologies are fundamentally predicated on the mechanical interaction between cutting tools or grinding implements and workpiece materials to achieve material removal. This methodology has been refined through decades of technological evolution, resulting in a mature processing system capable of delivering exceptional dimensional accuracy while maintaining compatibility with an extensive range of engineering materials. However, when machining materials with elevated hardness and superior strength, researchers have consistently observed constrained machining efficiency due to inherent limitations in material processability. In the traditional processing, residual stress, scratches and microcracks and other defects often occur on the surface of the workpiece, which jointly hinder the reduction of surface roughness parameters. Concurrently, the phenomenon of accelerated tool wear is widespread, which not only leads to an increase in production costs but also weakens the stability of machining accuracy. The limitations of traditional machining are evident in industrial cases: In aerospace, Inconel 718 turbine blades experience rapid tool wear and surface embrittlement due to heat accumulation during conventional milling, while Ti-6Al-4V compressor blades suffer from severe tool adhesion and poor surface roughness from traditional drilling [1]. In nuclear engineering, Alloy 600 steam generator tubes are prone to intergranular stress corrosion cracking (leading to coolant leakage) through traditional TIG welding, and Zircaloy-4 fuel cladding develops non-uniform oxide films and hydrogen embrittlement from conventional stretching [2].

**Figure 1:**
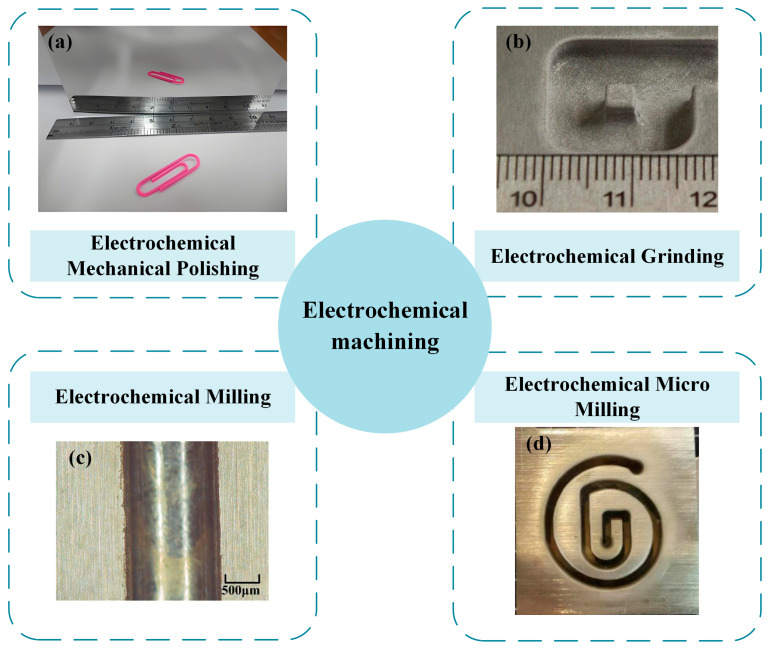
The result of electrochemical machining processing (**a**) Electrochemical Mechanical Polishing of stainless steel substrate. Reprinted from International Journal of Electrochemical Science, Copyright (2013), with permission from Elsevier [8], (**b**) Electrochemical Grinding of GH4169 alloy. Reproduced from Scientific Reports [9], (**c**) Jet Electrochemical Milling of Ti-6Al-4V alloy. Reprinted from Journal of Materials Processing Technology, Copyright (2019), with permission from Elsevier [10], (**d**) Electrochemical Micro-Milling of nickel-based alloy. Reprinted from Journal of Materials Processing Technology, Copyright (2020), with permission from Elsevier [11].

ECM is established as a material removal technology based on the principle of anodic dissolution. Under the influence of an applied electric field, oxidation reactions are induced at the anode to achieve controlled material erosion, while reduction reactions accompanied by gas evolution are simultaneously generated at the cathode. It has been established that this process exhibits several fundamental advantages [6,7]: its capability is entirely independent of the workpiece’s mechanical properties, making it exceptionally suitable for machining refractory metals; its operational efficiency is significantly enhanced through continuous ionic dissolution; it yields superior surface integrity characterized by the absence of mechanical residual stresses and a recast layer, as shown in Figure 1; and tool wear is effectively precluded by the inherent non-contact nature of the electrochemical process. Within this technological context, ECM has been progressively recognized as a critical advanced manufacturing technique within the non-traditional machining domain. Because its main goal is to improve surface quality and it has obvious advantages over traditional processing methods, the importance of this process has gradually increased in contemporary manufacturing research.

For instance, silicon carbide (SiC), as a core material of third-generation semiconductors, holds an irreplaceable strategic status in high-power electronic devices, high-frequency communication, and other fields. Its excellent properties such as wide bandgap, high breakdown field strength, and thermal conductivity impose the requirement that the surface must be in an atomic-level smooth state free of scratches and damage. However, due to the high Mohs hardness and strong brittleness of SiC, traditional mechanical processing easily induces deep scratches, subsurface damage, and residual stress on the surface, failing to meet the strict requirements for device fabrication. Through electrochemical-mechanical composite processing, the surface of SiC can be converted into a soft silicon dioxide layer via electrochemical oxidation; this layer is then mechanically removed to achieve high-efficiency precision processing. This approach avoids the defects of traditional mechanical processing and significantly improves surface quality, thereby providing key technical support for the application of SiC in high-end fields. In the research of electrochemical processing of silicon carbide, Gao et al. [12] utilized electrochemical mechanical polishing technology. By controlling the elastic effect of PS/CeO_2_ core-shell abrasives and the dynamic removal balance of the oxide layer (SiO_2_) on the surface of SiC, a mirror-like finish with a surface roughness (Ra) as low as 0.449 nm was achieved on 4H-SiC materials. Ma et al. [13] used plasma electrolysis treatment to oxidize the surface of 4H-SiC into a soft oxide layer mainly composed of SiO_2_. By combining mechanical polishing with CeO_2_ abrasives, they achieved a mirror-like finish with a surface roughness (Ra) of 8.1 nm. Yang et al. [14] used a three-step slurry free electrochemical mechanical polishing technique to generate a soft oxide layer through electrochemical oxidation and combine it with mechanical removal, significantly improving the surface integrity of SiC wafers, eliminating subsurface damage, and achieving atomic level smooth surfaces. The aforementioned studies demonstrate that ECM of silicon carbide delivers quantifiable improvements over conventional processing methods. By combining electrochemical oxidation with mechanical polishing, subsurface damage was effectively suppressed, and atomic-level smooth surfaces were obtained simultaneously, confirming the effectiveness of this method in the precise manufacturing of SiC components in advanced semiconductor applications.

Titanium-based materials play a crucial role in aerospace components and medical implants due to their excellent specific strength, corrosion resistance and biocompatibility. The surface quality of these materials directly affects fatigue strength, wear resistance and cell affinity. However, traditional mechanical polishing is limited by the high plasticity and strong adhesion of titanium alloys, which can easily lead to abrasive adhesion, work hardening and surface scratches, and at the same time, it is difficult to achieve nano-level surface roughness. Electrochemical mechanical polishing has emerged as a breakthrough solution. Electrochemical mechanical polishing converts the titanium surface into an easily removable TiO_2_ soft layer through electrochemical oxidation and combines it with mechanical polishing to achieve a synergistic “oxidation-removal” treatment. This method not only reduces surface hardness through electrochemical action, but also precisely controls the removal of the oxide layer through mechanical action, avoiding traditional processing damage. At the same time, it significantly improves surface flatness and processing efficiency, providing a revolutionary solution for the precision processing of titanium-based materials. For example, Tsuji et al. [15] used electrochemical mechanical polishing technology to generate TiO_2_ oxide layer on the surface of titanium and titanium alloys through electrochemical oxidation. Combined with the mechanical removal effect of SiO_2_ abrasive particles, a mirror-like finish was achieved, with surface roughness (Sa) values of 0.66 nm for pure titanium and 0.5 nm for Ti-6Al-4V alloy. Lee et al. [16] investigated the electrochemical mechanical polishing technique, whereby mechanical stress was reduced and oxide layer was controlled via non-contact processing. This approach enabled the achievement of no work hardening and enhancement of chemical stability, thereby improving surface integrity and fatigue life. These experimental findings demonstrate that electrochemical mechanical polishing offers clear advantages over traditional methods for processing titanium-based materials. The results of these studies verify that electrochemical mechanical polishing is a more efficient and precise method for finishing titanium alloys, providing a basis for its increasing popularity in high-performance applications.

### 1.2. Classification System of ECM Technologies for Surface Treatment

To address the surface requirements of advanced components in high-precision manufacturing, electrochemical surface treatment technologies can be systematically organized according to their primary objectives into two distinct pathways: “surface quality enhancement” and “material shaping”. This framework emphasizes the implementation routes aligned with technical goals, rather than imposing rigid categorization, thereby better reflecting the synergistic and interdisciplinary nature of industrial applications.

#### 1.2.1. Technological Pathways for Surface Quality Enhancement

This pathway is dedicated to optimizing surface integrity to meet performance requirements under specific operating conditions. For instance, in fields such as biomedical implants and biosensors, where superior biocompatibility and homogeneous surfaces are critical, the focus is on actively modifying the surface state through methods such as electrochemical anodic oxidation dissolution. These techniques effectively eliminate surface defects and residual stresses introduced by conventional machining, achieving nanoscale planarization. A targeted strategy for enhancing surface functionality, including biological performance, is formed by techniques that combine electrochemical dissolution with mechanical abrasion, as well as those utilizing pulsed currents for minimizing defects and refining surface topography.

The research approach aimed at optimizing surface quality can be supported by the following studies: For quenched and tempered steel (such as 42CrMo4), with electrolyte regulated electrochemical processing (ECM) as the core, the reduction of flow marks and the control of passivation layer have been achieved [17]; For the research on hard and brittle materials (such as 4H-SiC), electrochemical mechanical polishing (ECMP) is the key, which strikes a balance between SiC corrosion and SiO_2_ removal, achieves a scratch-free surface (Ra = 0.449 nm), and improves the processing efficiency (~2.3 μm/h) [12]; For the research on titanium alloys (pure titanium, Ti-6Al-4V), sub-nanometer roughness (pure titanium Sa = 0.66 nm) was achieved using ECMP, and grain boundary steps were eliminated [15].

#### 1.2.2. Multi-Scale Technological Pathways for Material Shaping

This direction focuses on achieving specific geometries and structures through material removal, and can be naturally distinguished into macro-scale and micro-scale approaches based on feature dimensions:

(1) Macro-Scale Material Shaping

In sectors such as aerospace and energy equipment, balancing high material MRR with micron-level precision is a primary requirement. Technologies that integrate electrochemical dissolution with conventional mechanical methods have been developed for this purpose. By optimizing the synergistic effects between electrochemical and mechanical actions, these methods enable effective control over workpiece macrotopography, dimensional tolerances, and structural stability, thereby unifying processing efficiency with precision.

The significance of this technical approach has been verified through application in the processing of macroscopic components of difficult-to-machine alloys. When studying the electrochemical turning technology for treating nickel-based superalloy rotating parts, the unification of a 10 mm radial removal allowance and a ±0.1 mm dimensional tolerance was achieved, confirming the core requirement of macroscopic forming for “high efficiency—high precision” [18]. In the study of macroscopic electrochemical milling through pulse voltage and electrode optimization, a high removal rate of 20~30 mm^3^/min and a flatness error of less than 8 μm were achieved in the processing of Inconel 718 alloy, further demonstrating the practical rationality of the macroscopic forming technology approach [19].

(2) Micro-Scale Material Shaping

The manufacturing challenges presented by hard and brittle materials at micro- and nano-scales are addressed through techniques that utilize masking layers and precision electrode design. These methods are dedicated to the precise fabrication of high-resolution microstructures. Processes such as through-mask electrochemical machining have been successfully implemented for creating three-dimensional microstructures in micro-electro-mechanical systems (MEMS) devices and optical components, thereby unifying manufacturing processes across significantly different dimensional scales and providing specialized solutions for micro-feature fabrication.

The technical approach for forming this microscale material can be supported by related research: mask-based electrochemical machining combined with side-wall insulating microtools can achieve microstructures with a resolution of 10 μm on hard and brittle materials such as 4H-SiC, confirming the effectiveness of mask layer and precise electrode design in the microfabrication of hard and brittle materials [20]. Mask electrochemical machining with pulsed power supply achieved mask pattern replication with a size error of less than 2 μm on monocrystalline silicon, further confirming the rationality of this technical approach for the cross-scale microstructure manufacturing of MEMS and optical components [6].

#### 1.2.3. Conclusions

Driven by component surface requirements, electrochemical technologies aimed at “enhancing surface quality”, “achieving macro-scale material shaping”, and “enabling micro-structure fabrication” collectively constitute a multi-dimensional technical system. Grounded in industrial needs, this system supports high-precision surface processing in a systematic manner. Through their capabilities in improving surface integrity and fabricating complex microstructures, these technologies significantly advance progress in high-tech fields including aerospace, semiconductors, and biomedicine.

## 2. Research Progress in Electrochemical Technology for Surface Processing

### 2.1. Surface Quality Improvement ECM Technology

Surface quality enhancement in electrochemical processing is defined by the achievement of surface integrity with nanometer to sub-micron accuracy. This level of accuracy is realized through controlled physicochemical interactions between the workpiece surface, the applied electrochemical field, and the electrolyte. The process is directed toward three core objectives: the reduction of surface roughness, the optimization of micro-topography, and the enhancement of functional properties. By implementing coordinated process control strategies, electrochemical dissolution and surface modification mechanisms are systematically combined to significantly improve overall surface integrity. A methodical approach based on electrochemical reactions is employed to eliminate traces of prior mechanical processing and improve critical surface performance metrics. Representative implementations of this methodology include pulsed electrochemical polishing and electrochemical mechanical polishing, which are designed to address specific surface engineering challenges and provide targeted solutions for diverse industrial applications.

#### 2.1.1. Pulsed Electrochemical Polishing

Technologies aimed at improving surface integrity play a crucial role in surface treatment techniques. As a frontier in this field, electrochemical surface treatment is committed to significantly enhancing surface integrity, among which reducing surface roughness and improving environmental compatibility are the key supporting directions for achieving this goal. Against this backdrop, electrochemical polishing has emerged as a key technology, and its ability to achieve nanoscale surface roughness on difficult-to-machine materials has made significant progress.

**Figure 2:**
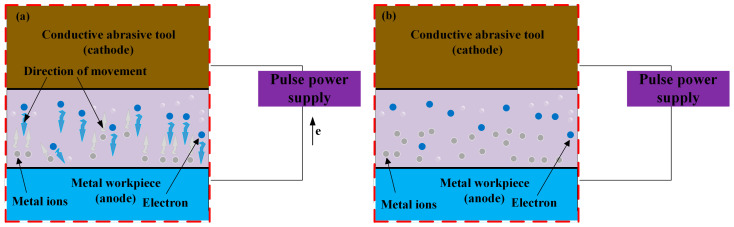
Schematic diagram of pulsed electrochemical machining (**a**) Polarization phase, (**b**) Relaxation phase.

**Figure 3:**
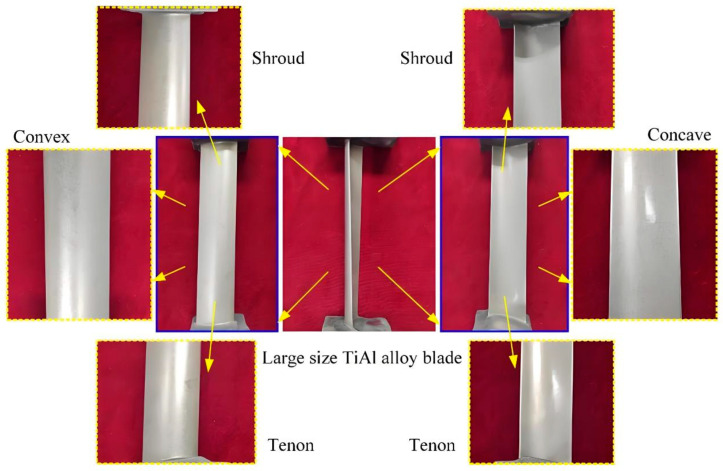
Large size TiAl alloy blade machined by pulse electrochemical machining. Reproduced from, Metals [21].

Pulsed electrochemical polishing is an improved form of electrochemical polishing, which uses a pulsed power supply to periodically activate the current to enhance the surface finish. The operating mechanism consists of two distinct electrochemical stages: the polarization phase, in which anodic oxidation efficiency is enhanced by instantaneous high current density, and the relaxation phase, during which electrolyte diffusion is promoted and concentration polarization is reduced. The principle is shown in Figure 2. Through this pulse control, three key improvements have been achieved: refinement of grain structure, minimization of processing defects, and enhancement of corrosion resistance. Therefore, pulsed electrochemical polishing has obvious technical advantages, such as significant improvement in MRR via pulse current regulation, optimized energy consumption via duty cycle control, and expanded applicability to different material types. The processing effect of this technology is shown in Figure 3 (The experimental results of Wang et al. [21]). Many researchers have also conducted in-depth studies on this electrochemical polishing method. For instance, in the direction of basic modeling and simulation of pulsed electrochemical mechanical polishing, Chen et al. [22] proposed a multi-physical field model and a quasi-state simplified algorithm to simulate temperature evolution and workpiece shape change in the pulsed electrochemical polishing process. Through time-averaged boundary conditions and heat sources, the calculation complexity is effectively simplified, and the accuracy of the model is verified by experiments. This study provides a theoretical framework for understanding the thermal effects and material removal mechanisms in pulsed electrochemical polishing.

In the research on workpiece materials and electrolytes, Chun et al. [23] studied the effect of different electrolytes, including NaCl, CH_3_COOH, NaNO_3_, and NaNO_2_ mixed with sodium tartrate, on the pulsed electrochemical machining processing performance of Invar sheet. It was found that the conductivity of the electrolyte is not simply directly related to the processability, but is dominated by the passivation capacity of the electrolyte ions. For example, ${\mathrm{NO}}_{3}^{-}$ and ${\mathrm{NO}}_{2}^{-}$ tend to form passive films. This study provides experimental basis for electrolyte selection. Wang et al. [24] analyzed the influence of pulse current parameters such as frequency, duty cycle and voltage on the surface morphology, profile accuracy and corrosion behavior of Inconel 718 pulsed electrochemical machining properties in NaNO_3_ solution. Short pulses and high frequencies were found to improve processing accuracy and reveal the nonlinear current efficiency characteristics of material dissolution. The two dimensions of electrolyte formula optimization and pulse parameter regulation provide theoretical basis and process guidance for efficient and precise ECM of alloys difficult to be machined.

In terms of process optimization and design, Fang et al. [25] proposed a cathode optimization method based on iterative solution of multi physics field models. By introducing the gap correction factor ψ and adjusting the distribution of electrolyte conductivity, the current density was homogenized, ultimately reducing the machining error from 0.2 mm to 0.06 mm. This study provides an effective strategy for the design of complex surface cathodes. Zhang et al. [26] innovatively adopted bidirectional electrolyte flow technology, which periodically reverses the flow direction of the electrolyte to homogenize the average temperature and dissolution rate over time. Experimental results confirm that this method reduces the geometric error of the anode to 1/17 of the original value without sacrificing processing efficiency, establishing an innovative approach for long-gap pulsed electrochemical polishing. These two studies respectively provide theoretical support for cathode design optimization and technical solutions for long-gap process control.

In terms of control strategy, Boxhammer et al. [27] pioneered the application of linear time-varying model predictive control to pulsed ECM processes. By estimating the dissolution parameter in real-time and dynamically adjusting the current reference value, they achieved coordinated control of the interelectrode gap and current, effectively suppressing machining errors. This work established the foundation for intelligent control in pulsed ECM.

Within the domain of electrochemical surface quality enhancement, systematic investigations have been conducted into the material dissolution mechanism, thermal-electric flow field coupling behavior, and machining accuracy control in pulsed electrochemical polishing. These studies employ multidisciplinary modeling, electrolyte optimization, process innovation, and intelligent control strategies. The resulting insights provide theoretical foundations and engineering methodologies for achieving surface quality improvement on complex components, thereby accelerating the industrial implementation of this technology.

#### 2.1.2. Electrochemical Mechanical Polishing

Electrochemical mechanical polishing is classified as an established technique within electrochemical metalworking. This process combines electrochemical dissolution with precisely controlled mechanical action, thereby enhancing material removal efficiency and improving surface quality.

**Figure 4:**
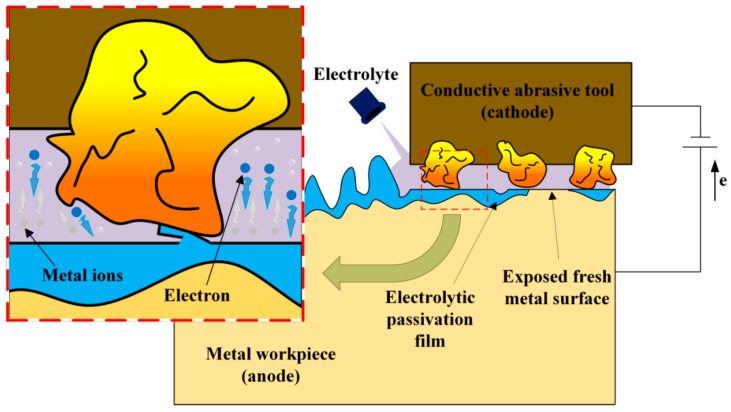
Schematic diagram of electrochemical mechanical polishing technology.

The operational principle of electrochemical mechanical polishing relies on synergistic electrochemical-mechanical interactions. Upon electric field application, oxidation reactions at the anodic workpiece achieve controlled material removal, while reduction reactions with gas evolution occur at the cathode. Anodic oxidation forms a passivation layer on the workpiece surface through reaction between dissolved metal ions and oxidizing species in the electrolyte. Subsequently, abrasive particles mechanically remove this passivation layer at surface protrusions, exposing fresh metal for continued electrochemical dissolution. Conversely, the passivation layer within recessed regions remains protected, thus preventing excessive erosion as illustrated in Figure 4. This coordinated process of electrochemical dissolution and precise mechanical wear contributes to the reduction of surface roughness.

The key influencing factors of this processing method include electrolyte properties, electrical parameters, mechanical parameters, material characteristics, and other factors. Many researchers have studied and analyzed this polishing method from different perspectives such as basic mechanisms and atomic scales, such as Zhu et al. [28] used ReaxFF molecular dynamics simulation to study the atomic scale process of electrochemical mechanical polishing of SiC in H_2_O_2_ solution, and summarized the synergistic effect of electric field enhanced oxidation and friction wear. Gao et al. [29] in situ monitored the competitive mechanism of oxide film formation and removal in tantalum CMP/ECMP (chemical–mechanical polishing/electrochemical mechanical polishing) using single-frequency electrochemical impedance spectroscopy to elucidate the synergistic effect of current density and mechanical power. Both of them provide a systematic understanding of the mechanism of electric field-mechanical coupling in electrochemical mechanical polishing technology from two dimensions of theoretical simulation and experimental monitoring, and lay a theoretical foundation for process parameter optimization and material surface integrity control.

**Figure 5:**
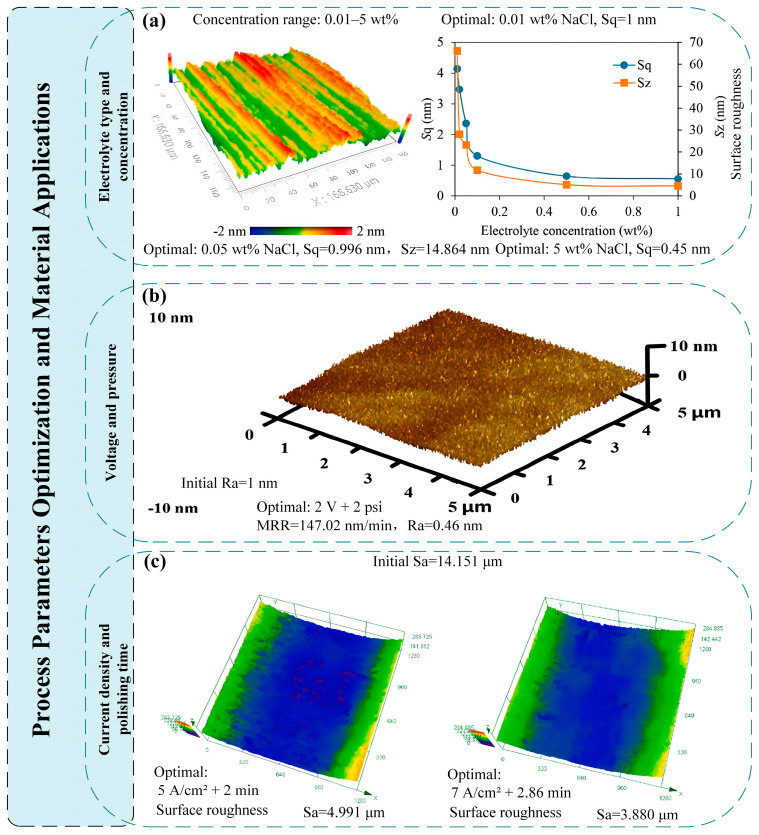
Effect of key process parameters on machining quality in electrochemical mechanical polishing, (**a**) Effect of electrolyte type (NaOH/NaCl/NaNO_3_) and concentration on ECMP quality of 4H-SiC (0001) wafers. Reprinted from Electrochimica Acta, Copyright (2024), with permission from Elsevier [30], (**b**) Effect of applied voltage and polishing pressure on ECMP quality of cobalt (IC barrier layer material). Reprinted from Materials Science in Semiconductor Processing, Copyright (2024), with permission from Elsevier [31], (**c**) Effect of current density and polishing time on ECMP quality of SLM 304 SS internal holes (Φ3 mm). Reprinted from Journal of Manufacturing Processes, Copyright (2021), with permission from Elsevier [33].

In the study of process parameter optimization and material application, Yang et al. [30] systematically investigated the influence of electrolyte type and concentration on the anodic oxidation of 4H-SiC slurry free electrochemical mechanical polishing, and found that conductivity is a key factor controlling the oxidation rate and uniformity. Xie et al. [31] determined the optimal parameters (2 V voltage, 2 psi pressure) of cobalt electrochemical mechanical polishing through orthogonal experiments. Under the optimal parameters, the MRR was 147.02 nm/min and the surface roughness was 0.46 nm. An et al. [32] achieved a synergistic effect of electrochemical dissolution and mechanical grinding by optimizing process parameters. Among them, the contribution rate of electrochemical dissolution to material removal was 72.2%, significantly improving the surface integrity of metal inner channels in additive manufacturing. The arithmetic mean deviation (Sa)/root mean square deviation (Sq) of the surface roughness of the 30 mm straight-through channel decreased by 71.2%/68% respectively, and the surface roughness of the 18 mm curved channel decreased by 79.9%/81.1% respectively. For the difficult-to-machine small-diameter channels with a diameter of 5 mm/9 mm, the arithmetic mean deviation (Sa) of surface roughness was significantly reduced from 15.92 µm/18.18 µm to 5.06 µm/6.02 µm. Zhao et al. [33] optimized the current density (2–7 A/cm^2^) and polishing time for the complex structure of 304 stainless steel material manufactured by SLM, achieving a reduction in surface roughness Sa from 14.151 µm to 3.880 µm. Hung et al. [34] significantly reduced the surface roughness (Ra) of the additively manufactured Ti-6Al-4V from 16 µm to 0.227 µm by optimizing electrochemical mechanical polishing parameters such as the polishing channel structure, cathode rotation/movement speed, and applied current, using a robotic electrochemical mechanical polishing process. At the same time, a dense passivation layer 10 nm thick is generated, enhancing corrosion resistance and increasing the absolute value of corrosion resistance (0.01 Hz) from 9788 Ω·cm^2^ to 27,292 Ω·cm^2^. These studies have improved the surface integrity of the workpiece after electrochemical mechanical polishing by optimizing process parameters such as material selection, electrochemical reaction control, and mechanical action balance. As shown in Figure 5, this optimization significantly improves the surface quality in various application scenarios, including semiconductor substrates, precision components, and biomedical implants.

In terms of environment-friendly technological innovation, Murata et al. [35] achieved the rapid flattening (MRR 15 µm/h) of the SiC surface by using a Nafion/CeO_2_ composite pad without liquid electrolyte, and the controllable removal of oxide layer was verified by XAFS analysis. Inada et al. [36] proposed the use of polystyrene sulfonic acid (PSS) solid electrolyte composite liners to achieve efficient polishing of 4H-SiC wafers, which not only achieved a MRR of 14.3 µm/h, but also avoided the environmental burden of traditional liquid electrolytes. Through the innovative design of solid electrolyte, they not only maintain polishing efficiency, but also eliminate waste liquid pollution, providing an important technical paradigm for green manufacturing in semiconductor material processing field.

Electrochemical mechanical polishing achieves nanoscale planarization while maintaining high material removal rates through a precisely controlled oxide film formation and removal mechanism. The processing effectiveness is derived from the synergistic integration of electrochemically enhanced oxidation and selective mechanical abrasion. This hybrid approach enables successful processing of challenging materials including SiC, tantalum, and GaN, while demonstrating environmental advantages through solid-electrolyte composite pads that significantly reduce waste generation. The technology further maintains consistent performance on complex geometric surfaces. Current research addresses persistent challenges in electric field distribution uniformity, process stability assurance, and multi-scale modeling accuracy. Future developments are directed toward advanced electrolyte systems, intelligent control methodologies, and extreme-condition processing capabilities to expand applications in high-end manufacturing sectors.

### 2.2. Macroscopic Material Forming ECM Technology

Macroscopic material forming by electrochemical methods is characterized by substantially higher material removal rates compared to surface integrity-oriented processes, while maintaining precision at micron to sub-micron scales. This class of technologies operates through coordinated electrochemical and mechanical interactions to achieve controlled material removal and accurate surface formation. Specific processes including electrochemical grinding, electrochemical milling-grinding, electrochemical milling, and electrochemical turning are employed to regulate surface morphology, modify functional properties, and implement precision corrections on complex components. These established methods enable efficient processing of difficult-to-machine materials while ensuring dimensional accuracy and geometrical compliance in industrial applications.

#### 2.2.1. Electrochemical Grinding

**Figure 6:**
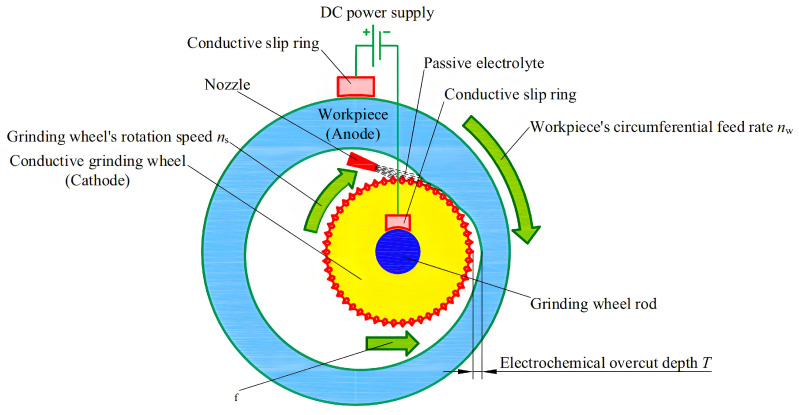
Schematic diagram of the internal cylindrical plunge electrochemical grinding process. Reprinted from Journal of Manufacturing Processes, Copyright (2023), with permission from Elsevier [42].

**Figure 7:**
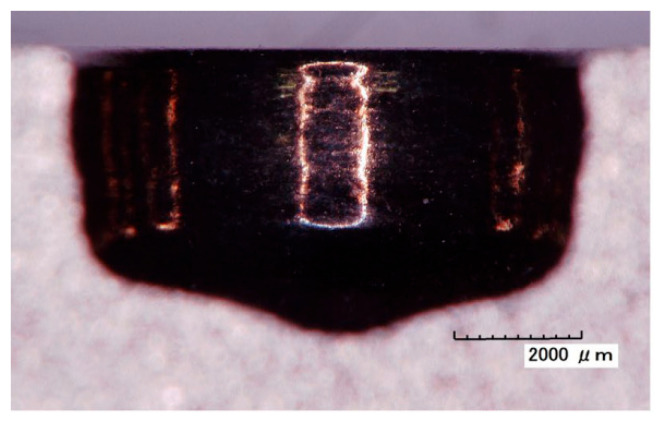
Electrochemical grinding cross-sectional processing effect drawing. Reproduced from Scientific [9].

Electrochemical grinding, an advanced precision technology, is characterized by the integration of electrochemical dissolution with mechanical abrasive processes, with primary applications in hard materials processing and high-efficiency, high-precision machining of materials such as ceramics, cemented carbides, and quenched steels. Its principle is to use a conductive grinding wheel with a metal binder serves as the cathode, while the workpiece acts as the anode. Under the action of a direct current electric field, electrochemical dissolution occurs on the surface of the workpiece to form a passivation layer. Meanwhile, the abrasive grains on the grinding wheel continuously remove the passivation layer through mechanical action, not only exposing the fresh base material to participate in the electrolytic reaction, but also improving the surface morphology of the workpiece. In this process, electrochemical dissolution removes the majority of material, while mechanical grinding eliminates the passivation layer and performs minor corrections, significantly reducing wheel wear and machining forces. The schematic diagram of this technology is shown in Figure 6. This method has the advantages of high efficiency, low wear, small residual stress and strong adaptability to complex geometries. The processing effect of this technology is shown in Figure 7. Due to its unique advantages in precision machining, extensive research has been conducted. For example, in terms of basic process parameters and material removal mechanisms, Xue et al. [37] investigated the influence of electrolytic processing parameters such as voltage on material removal and abrasive wear during grinding, and found that high-voltage electrochemical grinding could reduce abrasive wear and maintain a high MRR. They also established a grinding model to explain the mechanism of the parameters’ action. Li et al. [9] optimized the electrolyte outlet structure through simulation for the internal injection electrochemical grinding of GH4169 alloy, and experimentally verified the effects of voltage, electrolyte temperature, and pressure on the maximum feed rate and MRR, achieving a single 3 mm deep machining. These studies have deepened our understanding of material removal behavior from the perspectives of mechanism modeling and process optimization.

In terms of process improvement and composite processing technology, Yehia et al. [38] proposed a composite electrochemical grinding process by adding Al_2_O_3_ abrasive to the electrolyte. The research found that adding 5 wt% Al_2_O_3_ can significantly enhance the MRR and reduce surface roughness. At the same time, the mechanism of the abrasive’s effect on the current density distribution and the formation of the passivation film was clarified. Niu et al. [39] designed a bottom insulated grinding tool structure and optimized the surface flatness of GH4169 alloy processed by electrochemical grinding through simulation and experiments. They found that the insulating layout could reduce electrochemical overcutting and improve processing accuracy. The above-mentioned research has expanded the application potential of composite electrochemical grinding from the perspectives of material modification and structural optimization.

In some new applications and special scenarios, researchers have also conducted research, such as Li et al. [40] comparing two types of internal jet electrochemical grinding tools, and verifying the influence of tool structure on the uniformity of machining gap and surface quality through simulation and experiment, achieving high-precision control of GH4169 alloy planar machining. Wang et al. [41] innovatively applied electrochemical grinding for adaptive grinding of silicon anode materials, using MgH_2_ as an auxiliary abrasive to optimize silicon particle size through volume expansion stress and improve battery cycling stability.

In terms of theoretical modeling and numerical simulation, Ma et al. [42] established a MRR model for internal cylindrical plunge electrochemical grinding; combined with an equivalent plane grinding model, they analyzed the contribution ratios of voltage and feed rate to electrochemical dissolution and mechanical grinding, and elucidated the control mechanism of over cutting depth.

In terms of surface integrity and mechanical properties, Wang et al. [43] studied the surface microstructure and in-situ tensile properties of Ti-6Al-4V alloy after electrochemical grinding, and found that the surface roughness was significantly reduced under 300 V voltage, while the material strength and elongation were improved. Cracks originated from surface micro defects and propagated along slip lines. These investigations systematically examined electrochemical grinding for precision machining through three principal research dimensions: parameter optimization, composite technology development, and theoretical modeling. The influence mechanisms of voltage parameters, electrolyte composition, and tooling configurations on MRR, surface quality, and machining accuracy were elucidated through comprehensive experimental and simulation-based methodologies. Innovative process strategies, including abrasive particle admixture, internal jetting mechanism and insulating tool rest design, have been successfully applied to refractory materials such as GH4169 superalloy and special applications such as complex curvatures and thin-walled structures. These advancements have established a powerful theoretical framework and provided practical engineering solutions for enhancing the processing efficiency and surface integrity of precision manufacturing systems.

#### 2.2.2. Electrochemical Milling-Grinding

**Figure 8:**
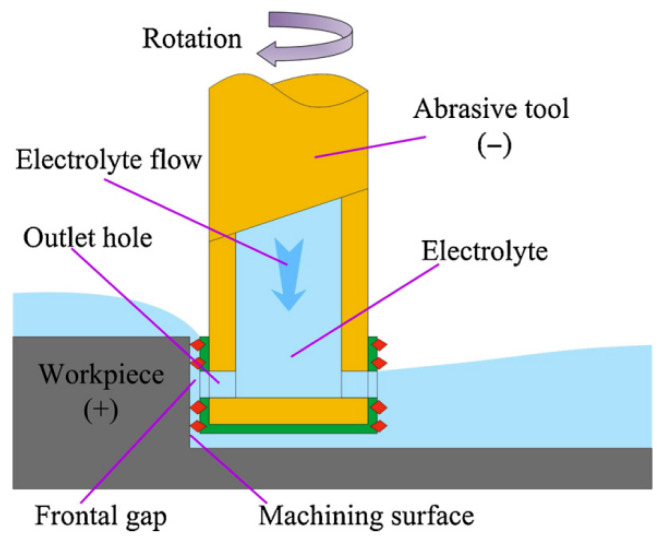
Schematic diagram of the electrochemical milling and grinding principle. Reprinted from Journal of Manufacturing Processes, Copyright (2019), with permission from Elsevier [44].

Electrochemical milling-grinding and electrochemical grinding share fundamental operational principles, with both methodologies categorized as high-efficiency precision machining technologies. However, the uniqueness of electrochemical milling-grinding lies in its hybrid process architecture, which systematically integrates milling and grinding processes. In this configuration, bulk material removal is predominantly executed by rotary cutting tools, which simultaneously enable complex structural machining capabilities. The schematic diagram of this technology is shown in Figure 8.

**Figure 9:**
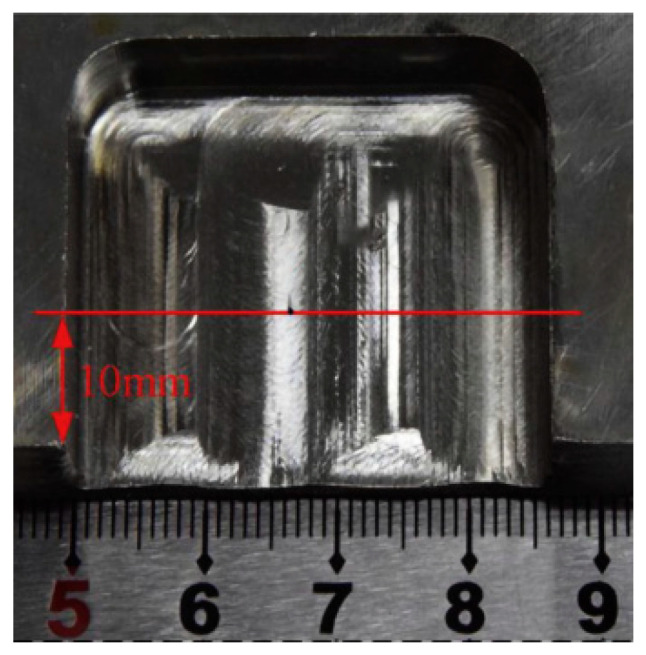
Electrochemical milling and grinding processing effect drawing. Reprinted from Journal of Materials Processing Technology, Copyright (2019), with permission from Elsevier [46].

This technology has greatly expanded the application scope of electrochemical processes, especially demonstrating high adaptability in the processing of comprehensive rough machining procedures. This adaptability is evident in the machining of complex three-dimensional geometries with substantial material allowances within the aerospace and renewable energy sectors. Consequently, electrochemical milling-grinding has emerged as a focal point of academic research, with systematic investigations conducted by numerous researchers to address multi-physics interactions and industrial implementation challenges. For instance, researchers from Nanjing University of Aeronautics and Astronautics conducted a comprehensive study on this technology in the context of tool design and flow field optimization, among whom Niu et al. [44] compared tools with different numbers of side holes and found that the four row side hole tool achieved the highest MRR (216.6 mm^3^/min) in Inconel 718 processing, and proposed the use of spiral side holes to improve sidewall flatness. Later, they [45] proposed a five-row side-hole grinding tool design for Ti-6Al-4V alloy, achieving efficient rough machining with a removal rate of 248.3 mm^3^/min for the alloy material and precise finishing with a surface roughness (Ra) of 1.06 µm. Based on this work, Yue et al. [46] designed a bottom additional outlet hole tool, which increased the flow velocity at the bottom edge of the groove through dynamic flow field simulation, reduced concave defects, and improved the flatness of Inconel 718 groove bottom by 61.2%. The processing effect is shown in Figure 9. On the basis of the above studies, Li et al. [47] optimized the layout of spiral side holes through flow field simulation, improved the uniformity of electrolyte distribution, reduced surface flow marks on Ti-6Al-4V machining, and achieved high-precision machining. The above series of studies have constructed a complete technological path from tool structure innovation to precise flow field control, providing a systematic solution for efficient and precise machining of complex three-dimensional structures.

In terms of material properties and mixing processes, Wang et al. [48] proposed a photocatalytically assisted electrochemical milling mechanism, which achieved the synergistic removal of SiC_P_/Al composites through aluminum-based dissolution and post-sic photocatalytic grinding, reducing the surface roughness Ra from 5.9 µm to 3.8 µm. Niu et al. [49] designed a bottom exit hole tool for titanium matrix composite. When processing titanium matrix composites with this bottom exit hole tool, the flatness of the groove bottom was improved by 93.4% in the finishing stage compared to the rough machining stage with the same tool. The surface roughness Ra reaches 0.37 µm. These two studies provide valuable solutions for efficient and precision machining of composite materials from the perspectives of process principle innovation and tool structure optimization.

In terms of process integration and surface quality, Qu et al. [50] reviewed macroscopic electrochemical milling and hybrid processes of electrochemical milling such as discharge milling and mechanoelectrochemical milling, emphasizing their advantages in the processing of complex thin-walled structures in aerospace. These studies have elucidated the efficient and precise machining mechanism of electrochemical milling and grinding technology in titanium alloys, nickel based alloys, and aluminum/titanium based composite materials through optimizing tool structures, multi physics field simulations, and hybrid processes, providing theoretical support and process innovation for the integrated manufacturing of complex thin-walled structures in aerospace.

#### 2.2.3. Electrochemical Milling

Electrochemical milling is characterized as a hybrid manufacturing process for precision machining that achieves stress-free, micron-level precision in complex three-dimensional structures on difficult-to-machine materials such as titanium alloys and ceramics. This ability is achieved through electrochemical anodic dissolution guided by a reaction controlled by tools. The process employs pulsed power supply systems coupled with precise electrolyte regulation to enable controlled material removal. The characteristics of electrochemical milling are three main advantages: eliminating mechanical cutting forces, improving dimensional accuracy, and adaptability to complex geometric structures. Consequently, comprehensive research investigations have been conducted across multiple technical dimensions, For example, in terms of material processing characteristics, Mishra et al. [51] compared the electrochemical milling performance of Ti-6Al-4V alloy with NaCl, NaBr, and NaNO_3_ mixed electrolytes through experiments. They found that NaCl (0.5 M) + NaNO_3_ (0.5 M) mixed electrolyte had the best machining accuracy and surface quality, and the optimization of feed rate and frequency could significantly improve machining depth and surface smoothness, breaking through the limitations of complex electrode designs in traditional ECM. Wang et al. [52] developed a novel electrochemical milling process to embed bismuth nanoparticles into a three-dimensional porous carbon framework (EMP-Bi@3DCF). This material has achieved ultra-high rate performance and long cycle life in sodium/potassium ion batteries at −20 °C, providing a new strategy for low-temperature battery materials. Niu et al. [53] designed a rotating electrode with a dead end tube and optimized the number of electrolyte outlets through flow field simulation. A high feed rate of 2.1 mm/min and a single processing depth of 3 mm were achieved in the internal spray ECM of nickel based alloy GH4169, successfully processing thin-walled structures and verifying the efficiency and stability of the process in difficult to process materials. Mishra et al. [54] used an internal spray rotating tool to study the electrochemical milling of Nimonic-263 alloy and found that a mixture of NaCl (1 M) and NaNO_3_ (1 M) electrolytes could achieve a super smooth surface with Ra of 0.06~0.08 µm. They also found that tool rotation could effectively reduce flow marks and stray corrosion. The optimal parameter combination obtained from the research (feed rate of 8 mm/min and layer depth of 0.3 mm) brings the processing depth close to the theoretical value (1.194 mm). Kong et al. [55] proposed jet electrochemical milling using ultra-high current density (200~600 A/cm^2^). Through multi-field coupling simulation and experimental verification, it was found that when the current density was ≥200 A/cm^2^, the electrolyte separated from the workpiece, which avoids stray corrosion. High MRR (282.9 mg/min) and non-corrosive edge were achieved on Ti-6Al-4V alloy, breaking through the accuracy limitations of traditional jet electrochemical milling. Liu et al. [56] first demonstrated the machinability of TB6 titanium alloy in a 20% NaNO_3_ solution. By elucidating the mechanism of rapid oxide film rupture under a high current density (140 A/cm^2^), they successfully machined grooves and flat surfaces. The resulting surface roughness was significantly better than that achieved under a low current density (40 A/cm^2^). This work provides a new method for the precision machining of aerospace titanium alloys. These studies systematically elucidate the machining characteristics of electrochemical milling in difficult to machine materials from the perspectives of electrolyte optimization, tool design, nanostructure regulation, and ultra-high current density applications, promoting the engineering application of this technology in fields such as aviation and energy.

**Figure 10:**
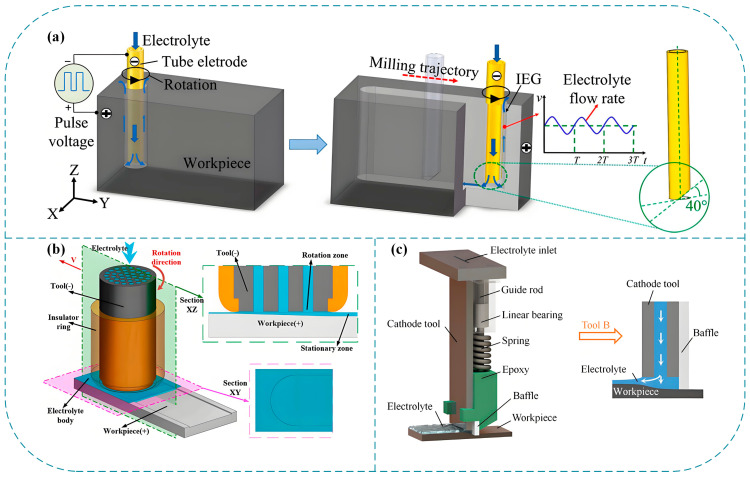
Cutting—Edge Tools, (**a**) wedge shaped end face tube (WS-tube). Reprinted from CIRP Journal of Manufacturing Science and Technology, Copyright (2022), with permission from Elsevier [57], (**b**) multichannel cathode. Reprinted from Journal of Manufacturing Processes, Copyright (2024), with permission from Elsevier [58], (**c**) backward parallel flow tool. Reproduced from Chinese Journal of Aeronautics [59].

In terms of process optimization and flow field control, Chen et al. [57] designed a wedge-shaped end face tube to enhance mass transfer in the deep narrow groove machining gap through pulsating flow field. Experiments have shown that compared to traditional flat end face tube, wedge-shaped end face tube tube increases the maximum feed rate from 0.24 mm/min to 0.42 mm/min, significantly reduces groove width errors and taper, optimizes surface roughness (Ra) to 0.7 µm, and successfully processes high-quality deep narrow grooves with a depth of 5 mm and a width of 1.48 mm. Shen et al. [58] proposed a multi-channel rotating cathode that adjusts the current density distribution through a bottom porous structure. Experimental verification shows that this design reduces surface straightness error (Δh) and depth error (Hde) by 80.9% and 69.4%, respectively. Under optimized parameters (voltage 25 V, speed 500 rpm), 42 × 40 mm flat machining was achieved with a flatness index (Δhp) of only 0.047, significantly improving machining efficiency and surface quality. Kong and Qu [59] developed a tail parallel flow adjustment tool to isolate the electrolyte in the processed area through an elastic baffle, eliminating weak current density areas in TC4 alloy flat processing. The experimental results showed that the surface roughness (Sa) decreased from 3.75 µm to 0.37 µm, and the MRR increased by 93–163%, while avoiding local corrosion caused by hydrogen bubble accumulation and maintaining the inherent hardness of the material. Three studies have achieved the engineering goals of improving the efficiency of deep and narrow groove machining, optimizing the machining accuracy of complex structures, and improving the surface quality of difficult to machine materials through innovative tool structures and dynamic flow field control, as shown in Figure 10.

In terms of processing mechanism and composite technology, Van Camp et al. [60] mechanically removed the passivation layer of ECM by rotating tools. Compared with the independent ECM with enhanced stability, the removal rate of titanium alloy materials was increased by 11%. The resulting roughness is approximately 5 μm, which is suitable for rough machining, but surface optimization is required. Kong and Qu [61] proposed an ECM-ECDM (Electrochemical Discharge Machining) composite mode for electrochemical milling by adjusting the electrode gap. In this mode, ECDM increases the MRR to 564% in the front zone, and ECM eliminates the recast layer in the rear zone, achieving a surface roughness (Sa) as low as 0.424 µm, breaking the bottleneck of precision machining of difficult-to-cut materials. Wang and Qu [62] classified the mechanical-electrochemical milling processing of TC4 titanium alloy, namely the mode, into three categories based on the relationship between the feed rate v_f_ and the first critical feed rate v_ff_ and the second critical feed rate v_sf_: When the feed rate v_f_ is less than or equal to v_ff_, it is a pure electrolytic processing mode, and only electrochemical dissolution occurs; When v_f_ is between v_ff_ and v_sf_, it is an electrolytic-mechanical-electrolytic compound mode, and electrolytic pre-processing, mechanical milling and electrolytic finishing are carried out in sequence in a coordinated manner. When v_f_ is greater than or equal to v_sf_, it is a mode mainly based on mechanical cutting and supplemented by electrolytic finishing. Research shows that processing within the critical speed range can significantly increase the MRR, and the final surface quality is mainly determined by the electrolytic finishing step. Parameter optimization is needed to reduce residual scratches. These studies provide theoretical basis and technical support for improving the processing efficiency and surface quality of difficult to machine materials through innovative composite processing modes and critical parameter regulation.

In terms of surface quality and accuracy control, Yu et al. [63] proposed a multiphysics field-coupled error compensation method for machining gap control based on current feedback, which dynamically adjusts the electrode position by collecting real-time machining current fluctuations and designing end effectors. Experiments have shown that this method controls the machining gap fluctuations caused by robot trajectory errors within 4% of the initial gap, optimizes the surface roughness (Ra) to 0.24 µm, and significantly improves machining accuracy and surface quality. Wang et al. [10] eliminated the low current density area in the edge region of jet electrochemical milling by adjusting the jet angle θ to 0°. The experiment successfully prepared a deep groove with a depth of 1.208 mm, with sharp edges and no stray corrosion. The surface roughness (Ra) was reduced to 0.352 µm, breaking through the limitations of poor edge quality in traditional jet ECM. Zhang et al. [64] designed an insulated front rectangular cathode tool to optimize end-region current density distribution. This tool reduced surface roughness (Ra) from 0.75 µm to 0.42 µm for 316L stainless steel, decreased overcut area by 40%, and achieved mirror finishing with surface roughness (Ra) of 0.24 µm, demonstrating significant quality enhancement through structural design. These studies have significantly improved the machining accuracy and surface quality of electrochemical milling through tool innovation, flow field regulation, and multi physics coupling models, providing efficient solutions for precision manufacturing of difficult to machine materials and having important application value in fields such as aerospace.

Electrochemical milling has been established as a hybrid manufacturing methodology that achieves stress-free, high-precision formation of complex geometries in difficult-to-machine materials through controlled electrochemical dissolution guided by tool-directed reactions, supported by precise regulation of pulsed power supplies and electrolyte systems. Significant advancements have been achieved through four principal research directions: optimization of electrolyte formulations, development of rotational tool systems, implementation of high-current-density processing protocols, and establishment of multi-physics coupling model frameworks. Systematic improvements have been realized in three critical performance domains: enhancement of MRR, refinement of surface roughness control, and improvement of deep narrow slot machining accuracy. Furthermore, substantial engineering application potential has been demonstrated through the integration of hybrid processing strategies with real-time gap compensation techniques, particularly in precision manufacturing of aerospace thin-walled structures and next-generation energy material components. This technological progression effectively addresses the convergence of theoretical modeling and industrial implementation constraints.

#### 2.2.4. Electrochemical Turning

**Figure 11:**
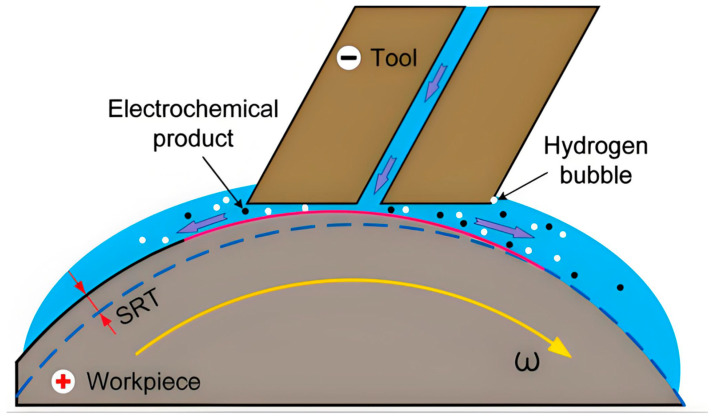
Schematic diagram of flow field distribution and material removal with inclined cathode tool during counter-directional machining. Reprinted from CIRP Journal of Manufacturing Science and Technology, Copyright (2025), with permission from Elsevier [66].

Electrochemical turning is recognized as a non-contact precision machining methodology in advanced manufacturing technologies, which is fundamentally based on the principle of electrochemical anodic dissolution. The operational mechanism employs direct current voltage between a rotating anode (workpiece) and a stationary cathode (tool electrode), achieving material removal through synergistic electrochemical dissolution and electrolyte flushing. This process exhibits three core attributes: absence of mechanical stresses, high-efficiency shaping capability, and superior surface integrity. It demonstrates exceptional performance in machining rotationally symmetric components, enabling geometrically precise surfaces and efficient removal of moderate-to-high material allowances with minimal mechanical stress. Given these technical advantages, researchers have conducted systematic investigations through multidisciplinary approaches to elucidate underlying mechanisms and optimize processing parameters, such as Yahyavi Zanjani et al. [65], who achieved gap control in electrochemical turning through tubular electrodes and Wheatstone bridge sensors. Experiments have shown that the MRR increases with voltage and time, decreases with the increase of gap, and the surface roughness increases with the increase of voltage. Ma et al. [66] optimized electrochemical turning of titanium matrix composites using a 60° tilted cathode design and flow field simulations (as shown in Figure 11) to enhance electrolyte flow. This resulted in a 167.1% increase in single-cycle removal thickness and a 63.5% reduction in surface roughness (Ra) for the composites. Ebeid et al. [67] combined electrochemical turning with roller burnishing, optimized parameters via the Taguchi method to eliminate pitting, achieving a improvement ratio of surface roughness of up to 81% and enhanced surface hardness. Liu et al. [68] optimized the jet electrochemical turning process through flow field simulation and successfully processed cylindrical, conical, and curved structures of TB6 titanium alloy, with a contour error of <1% and a surface roughness Ra of 2.414 µm. Ge et al. [69] designed an internal jet cathode to reduce stray corrosion during nickel-based alloy processing, successfully machining a 30 mm-high complex structure and verifying large-margin removal potential. Building on this, Ge et al. [70] adopted a universal cylindrical electrode with an internal flow field design, which can efficiently process various rotating structures such as cylinders, cones, and variable diameter components, with a radial removal allowance of up to 10 mm. These studies form a complete system from mechanism to application, providing solutions for precision machining of difficult to machine materials from multiple perspectives such as gap control, cathode structure optimization, hybrid technology, flow field simulation, and large margin machining.

Electrochemical hybrid processing technologies, which encompass electrochemical grinding, milling, combined milling-grinding, and turning operations, are characterized by the integration of controlled electrochemical dissolution with regulated mechanical actions. This integration enables efficient machining of difficult-to-cut materials with minimal subsurface damage. Implementation of these processes for aerospace structural components and advanced energy systems is facilitated through three critical technological developments: advanced tooling systems, precision electrolyte flow field control, and multiphysics simulation methodologies. This systematic approach effectively bridges theoretical modeling with industrial implementation, delivering solutions for manufacturing applications requiring micron-level accuracy and preserved material integrity.

### 2.3. Microscopic Material Forming ECM Technology

An acute contradiction has been formed between the urgent demand for precision microstructures in high-end fields and the inherent limitations of traditional machining (e.g., stress-induced damage, tool wear, and heat-affected zones). However, microscopic forming ECM based on anodic dissolution has emerged as a key solution, owing to its unique advantages of stress-free processing, thermal-damage-free material removal, and adaptability to difficult-to-machine materials. In this chapter, the mechanism innovations and research progress of six core technologies, namely Wire Electrochemical Turning, Electrochemical Micro-Milling, Electrochemical Micro-Turning, Wire Electrochemical Cutting, Mask Electrochemical Machining, and Jet Electrochemical Machining, are systematically elaborated.

#### 2.3.1. Wire Electrochemical Turning

**Figure 12:**
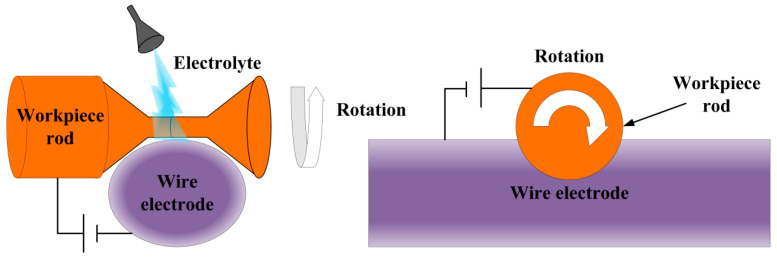
Schematic diagram of the principle of wire electrochemical turning.

Wire electrochemical turning differs from conventional methods through three key innovations: (1) a continuously wound movable thin-wire cathode eliminating tool wear, (2) bipolar pulsed current with neutral electrolytes enabling localized dissolution, and (3) enhanced versatility and environmental compatibility. The schematic diagram of this technology is shown in Figure 12. The electrochemical turning method for wires further demonstrates its high-precision processing capability with complex geometric shapes by enhancing the localization effect. For example, Han et al. [71] developed a wire electrochemical turning process combining neutral electrolyte and bipolar pulse current with wound stainless steel wire electrodes to eliminate tool wear. Their methodology successfully processed tungsten micro-rods with a minimum diameter of 11 µm and an aspect ratio of 36, breaking traditional processing limits. El-Taweel et al. [72] established a model based on the response surface method, clarified the interaction among parameters, and optimized the Settings of parameters such as voltage, feeding speed, and rotational speed, achieving a MRR of 0.298 g/min and a surface roughness (Ra) reduction of 1.13 µm. The roundness error (RE) has been reduced to 5.54 µm. Based on the above research, El Taweel et al. [73] further optimized the electrochemical turning parameters using fractional factorial design and found that voltage, electrolyte concentration, and overlap distance were significant factors. After optimization, the metal removal rate was increased to 187.75 × 10^−3^ g/min, verifying the effectiveness of the statistical experimental design. These researchers have gradually deepened the precision machining capabilities of wire electrochemical turning from the perspectives of process development, multi-objective modeling, and experimental design optimization.

Wire electrochemical turning has emerged as an advanced precision machining method by integrating a movable thin-wire cathode, bipolar pulsed current, and neutral electrolytes. This integration effectively resolves tool wear limitations in conventional electrochemical turning, enabling consistent micron-to-nanoscale precision. Current research systematically focuses on three domains: process parameter optimization, multi-physics modeling, and intelligent experimental design. The technique demonstrates significant advantages in microscale fabrication of difficult-to-machine materials, offering an innovative solution that combines environmental sustainability with submicron-level accuracy for high-precision manufacturing.

#### 2.3.2. Electrochemical Micro-Milling

Electrochemical micro-milling enables precision microfabrication through anodic dissolution at the ionic scale. Supplementary mechanical assistance enhances dissolution uniformity in micro-cavities. This approach eliminates mechanical stress and tool wear, making it ideal for micromachining brittle materials and high-strength alloys like titanium and nickel-based superalloys. Therefore, many researchers have conducted research on it, such as Zhang et al. [11] established a multi-physics coupling model, optimized tube electrode parameters such as pressure and voltage, and achieved single-channel manufacturing of blind grooves with a length-to-diameter ratio of 6.1. This breakthrough overcomes the efficiency limitations of traditional multi-pass processing. Mishra et al. [74] proposed a tool-rotation centrifugal flushing strategy to address micro-milling challenges in titanium/nickel/cobalt alloys, fabricating three-dimensional structures with aspect ratios >10 in Haynes-188 alloy and achieving a surface roughness (Ra) of 0.1 µm. Two studies have significantly improved the processing efficiency and surface integrity of complex structures through modeling optimization and process innovation.

**Figure 13:**
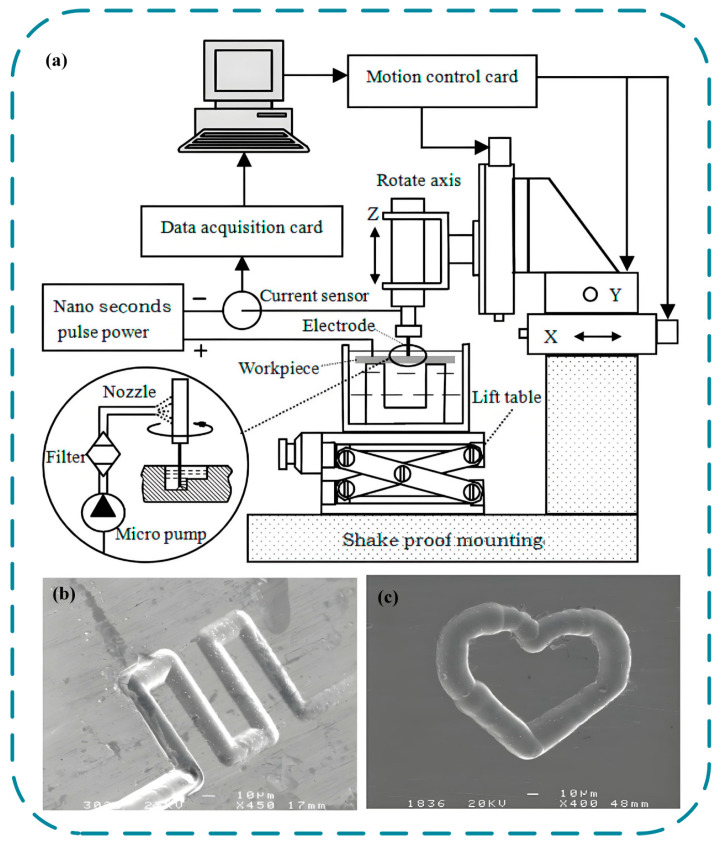
Processing principle and processing effect drawing, Reproduced from Micromachines [76], (**a**) schematic diagram, (**b**) processing effect drawing 1, (**c**) processing effect drawing 2.

Some researchers have been deeply involved in this field, such as Liu et al. [75] who developed in-situ preparation technology for 10 µm cylindrical electrodes. Through layer milling process, two-dimensional shapes with a width of 25 µm and three-dimensional stepped structures with a depth of 45 µm were successfully machined on GH3030 nickel based alloy, verifying the matching relationship between electrode diameter and layer thickness and providing key process parameters for microelectrochemical milling. On this basis, Liu et al. [76] established a positioning model based on nanosecond pulse power supply, and achieved high-precision machining with a side gap of only 25 µm and a surface roughness Ra as low as 0.125 µm by optimizing the voltage (4.5 V) and pulse width (95 ns), further verifying the advantages of short pulse parameters in improving machining positioning accuracy. The processing principle and effect of this technology are shown in Figure 13. To solve the efficiency problem of traditional ECM, Liu et al. [77] introduced a high-speed spiral electrode (20,000 rpm) to enhance the flow field, combined with simulation optimization parameters (5.5 V, 450 ns), and successfully machined a complex structure with a width of 150 µm on nickel based alloys, significantly improving machining efficiency and structural complexity, marking the team’s technological leap from basic processes to efficient machining. This research series established a comprehensive technical system spanning microelectrode preparation, pulse parameter optimization, and high-speed flow field enhancement. Marking a significant breakthrough in transitioning microelectrochemical milling from fundamental processes to engineering applications.

These investigations have systematically examined electrochemical micro-milling technologies for high-aspect-ratio structures, difficult-to-machine material compatibility, and electrode optimization through advanced methodologies. Conventional limitations in machining efficiency and precision have been addressed through three principal approaches: flow field intensification via high-speed electrolyte delivery, in-situ electrode preparation, and multiphysics simulation frameworks. This progress establishes a theoretical foundation and technical framework for precision manufacturing of complex metallic microstructures, specifically addressing challenges in microscale geometric fidelity and material-specific process adaptation.

#### 2.3.3. Electrochemical Micro-Turning

**Figure 14:**
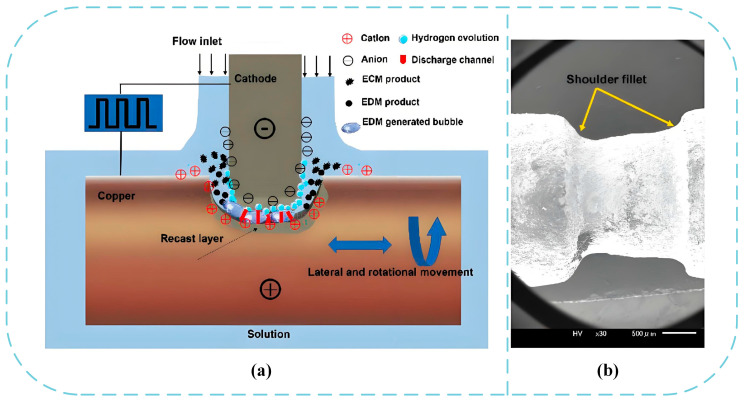
Electrochemical discharge turning diagram of the sandwich cathode structure. Reprinted from Precision Engineering, Copyright (2025), with permission from Elsevier [80], (**a**) principle diagram, (**b**) processing effect diagram.

Electrochemical micro-turning is a non contact microfabrication technology primarily utilizing electrochemical anodic dissolution, enhanced by rotational workpiece kinematics and optional auxiliary fields. The process applies pulsed voltages between a rotating anode (workpiece) and stationary cathode (tool) within electrolyte, inducing controlled material removal. Retaining core electrochemical advantages including negligible mechanical stress and submicron surface finishing, electrochemical micro-turning enables precise microscale feature generation on brittle materials and high strength alloys that are challenging for conventional machining. For example, Skoczypiec et al. [78] conducted experimental research on electrochemical assisted micro-turning of 1.4301 stainless steel and found that when the cutting depth is ≤1 µm, electrochemical assistance can reduce cutting force by 5–65% through surface passivation, verifying the potential of this technology in reducing mechanical stress and tool wear. On the basis of Skoczypiec et al., Grabowski et al. [79] further analyzed two electrochemical assisted variants, A and B. Research has found that variant B reduces cutting force by 5–65% and optimizes surface roughness Ra from 0.1–0.43 µm to 0.08–0.15 µm at a voltage of 3 V. This study clarifies the key role of surface passivation in suppressing plowing effects and proposes that this technology is suitable for the finishing stage of microfabrication, providing a theoretical basis for precision machining of complex microstructures. Wang et al. [80] proposed electrochemical discharge turning with a sandwich cathode structure. After optimizing the voltage (60–90 V) and electrolyte concentration (5–10 g/L), the over cutting amount of the micro shaft shoulder was reduced by 20%, and the surface roughness Ra reached 0.15–0.5 µm, breaking through the shoulder deformation problem of traditional turning, as shown in Figure 14, it is a comparison between the principle diagram and the processing effect diagram. These studies combine experiments and simulations to elucidate the mechanism of electrochemical assisted micro-turning in reducing cutting forces and optimizing surface quality through surface passivation, breaking through the traditional problem of shoulder deformation in turning and providing a low stress solution for complex metal microstructure machining.

#### 2.3.4. Wires Electrochemical Cutting

Wire electrochemical cutting employs electrochemical dissolution principles where a metallic wire cathode facilitates controlled anodic dissolution of the workpiece (anode) in electrolyte solution to remove material. This process eliminates thermal damage while enabling high-precision micro/nanoscale machining. For difficult-to-machine materials such as titanium alloys, NiTi, and tungsten, cutting efficiency and surface quality are improved through vibration-assisted debris evacuation, multi-wire configurations, and pulsed current optimization. These techniques are essential for achieving sub-micrometer feature resolution in micro-manufacturing. Industrial applications include aerospace, medical devices, and precision manufacturing. Given its advantages of no heat-affected zones, tool wear elimination, and compatibility with challenging materials, wire electrochemical cutting has been extensively studied from multiple research perspectives, such as basic process and electrode innovation. Zeng et al. [81] established a unidirectional moving wire flow field model, optimized the linear velocity and feed velocity parameters, and achieved uniform narrow slit processing of 5 mm thick stainless steel with a width deviation of ±15 µm. Debnath et al. [82] innovatively developed reverse etching combined with piezo-electric transducer vibration technology to prepare tungsten micro-wires with repeated cross-sectional changes (diameter 33 ± 1.5 µm), and the surface roughness Ra of wire electrochemical cutting processing was reduced to 0.09 µm. These studies provide efficient and stable solutions for microscale precision machining from the perspectives of flow field regulation and electrode preparation technology.

In terms of processing accuracy and surface quality optimization, He et al. [83] employed pulsed wire electrochemical cutting integrated with axial erosion technology to machine γ-TiAl alloy. Through optimization of the electrolyte and pulse parameters, they achieved a side gap of 109 μm on a 10 mm-thick workpiece and fabricated high-aspect-ratio structures reaching 31:1. Wu et al. [84] treated the surface of wire ECM with deionized water and optimized the electrode feed and movement distance, reducing the roughness of 304 stainless steel from 3.14 µm to 1.01 µm and eliminating the recast layer. Singh et al. [85] developed a sequential laser-electrochemical process for machining Inconel-718, where laser pre-cutting was followed by wire electrochemical cutting. Through parameter optimization, surface roughness was reduced from 6.03 µm to 0.86 µm, significantly improving surface finish with micron-scale kerf control and sub-µm surface integrity. These three studies have significantly improved the machining accuracy and surface integrity of difficult to machine materials through process parameter optimization, post-processing techniques, and composite processing modes, providing diversified solutions for the field of precision manufacturing.

**Figure 15:**
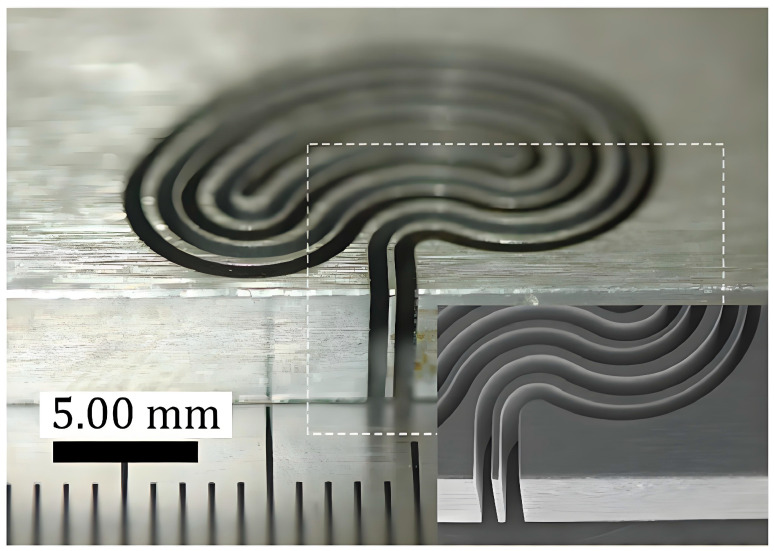
Image of the processing result of the vibratory ribbed wire tool. Reprinted from CIRP Annals - Manufacturing Technology, Copyright (2017), with permission from Elsevier [87].

In terms of auxiliary technology innovation, Zou et al. [86] proposed a vibrating ribbed wire tool, which promotes electrolyte circulation through a groove structure, improves dissolution positioning accuracy by 10%, and successfully processes 5 mm thick stainless steel barcodes. Fang et al. [87] from the same team introduced rib shaped wire electrodes with large amplitude vibration to enhance electrolyte renewal and bubble discharge, resulting in a 22% increase in MRR and achieving complex structural processing of 5 mm thick stainless steel. The processing result of this technology is shown in Figure 15. Klocke et al. [88] developed a rotating electrode combined with axial flushing technology to optimize electrolyte flow, increasing the cutting rate to 5.5 mm^2^/min and achieving a surface roughness Ra of 0.5 µm. These studies have significantly improved the positioning accuracy, material removal efficiency, and surface quality of ECM through tool vibration, structural optimization, and flow field control.

**Figure 16:**
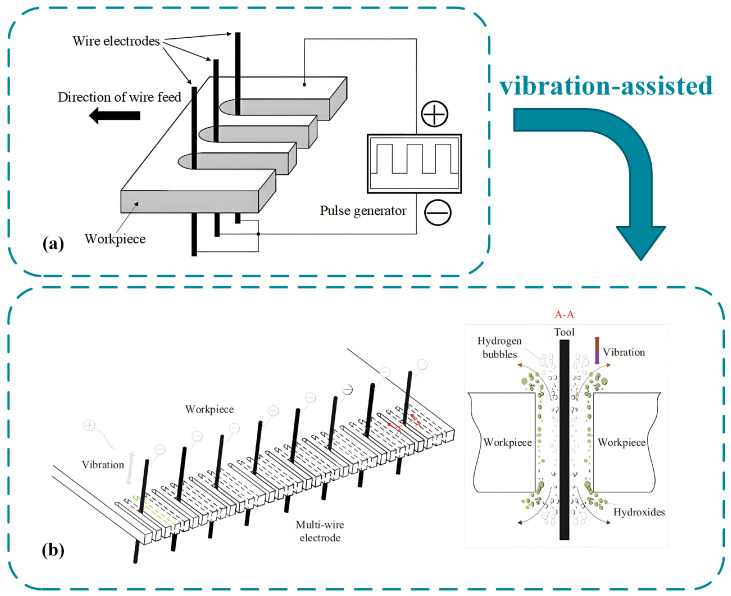
Comparison diagrams of multi-wire electrochemical machining with and without vibration, (**a**) schematic diagram of multi-wire electrochemical machining device. Reprinted from Journal of Manufacturing Processes, Copyright (2020), with permission from Elsevier [89], (**b**) schematic diagram of vibration-assisted multi-wire electrode machining. Reprinted from Journal of Manufacturing Processes, Copyright (2017), with permission from Elsevier [90].

In terms of multi line parallel machining and efficient strategies, Maity et al. [89] developed a multi-wire electrochemical machining system, which combined axial electrolyte flow and piezoelectric transducer vibration flushing strategy to achieve a high feed rate of 1.7 µm/s on stainless steel, and machined a micro slot array with a slot width of 90.62 µm and a standard deviation of 1.94 µm. He et al. [90] proposed an axial vibration-assisted multi-wire electrode method. By optimizing pulse parameters such as frequency and duty cycle, as well as vibration amplitude, the feed rate of 15 wire electrodes was 5.0 mm/s, achieving high-quality X-shaped part processing with a length-to-diameter ratio of 20 and a surface roughness Ra of 128 nm. This study demonstrates that axial vibration significantly enhances machining accuracy and complex structure formation, reflecting the iterative optimization of vibration-assisted strategies in multi-wire parallel machining, as shown in Figure 16 comparing multi-wire electrochemical machining processes with and without vibration.

In the application verification of complex materials and microstructures, Besekar et al. [91] utilized vibration-assisted axial jet line cutting technology to process nickel-titanium shape memory alloy in 0.1 M H_2_SO_4_ electrolyte, obtaining micro-slits with a width of 110 µm, a standard deviation of 0.57 µm, and a surface roughness Ra of 0.108 µm. The feasibility of wire cutting in the precision processing of biocompatible materials was verified. He et al. [92] achieved a microstructure with a slit width of 18 µm and an aspect ratio of 5.6 on pure tungsten by optimizing pulse parameters (5 V/60 ns/0.6 µs) and low concentration KOH electrolyte. They also used multi wire electrodes to increase efficiency by three times, providing a new method for X-ray grating manufacturing. Qu et al. [93] combined pulse ECM with reciprocating electrodes to machine a highly uniform microstructure with a seam width of 177 µm and an aspect ratio of 113 on stainless steel, verifying the effect of reciprocating motion strategy on improving machining accuracy. These studies have achieved high-precision microstructure machining of difficult-to-machine materials including γ-TiAl, pure tungsten, NiTinol, and stainless steel through innovative strategies such as vibration assistance, multi-wire parallelism, and pulse parameter optimization. This verifies the process advantages of wire electrochemical cutting in precision manufacturing while significantly expanding its engineering applications for complex materials.

#### 2.3.5. Mask Electrochemical Machining

Mask electrochemical machining is a technique where the electric field distribution is constrained by an insulating mask, such that anodic dissolution is confined exclusively to workpiece areas not covered by the mask. This enables the high-precision shaping of arrayed microstructures. Its core advantages include high localization accuracy, absence of tool wear, and suitability for batch processing. It is particularly well-suited for manufacturing arrayed structures such as MEMS devices, cooling holes in aero-engines, and fuel cell flow channels, addressing the limitations of traditional electrochemical micromachining in terms of efficiency and consistency for large-area array fabrication. The following sections elaborate on recent research progress from three aspects: process optimization, technological innovation, and the shaping of specific structures.

**Figure 17:**
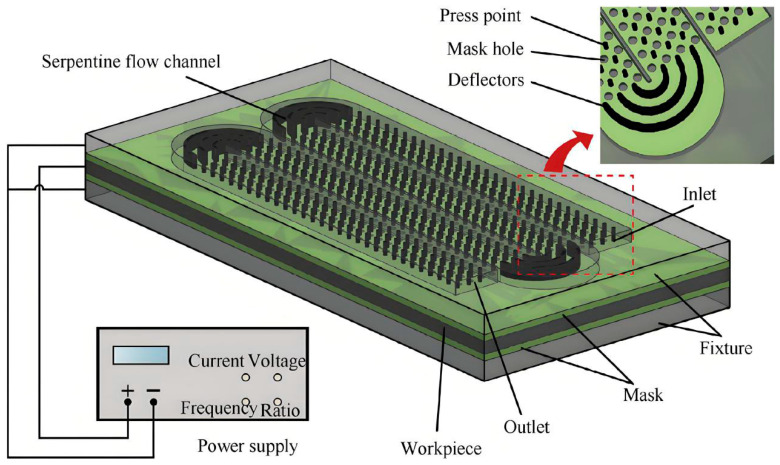
Schematic of the serpentine flow channel. Reproduced from Chinese Journal of Aeronautics [96].

**Figure 18:**
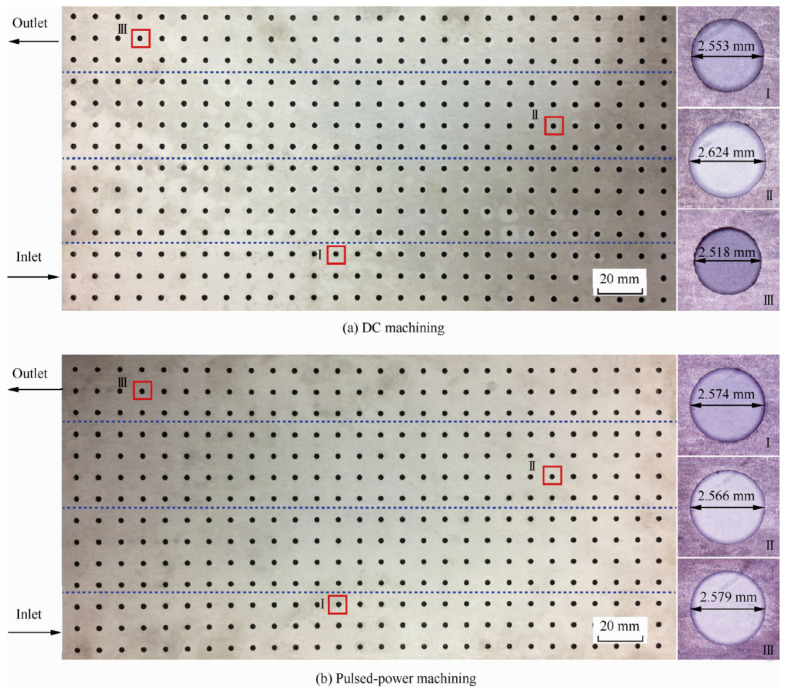
Photos and micrographs of hole arrays. Reproduced from Chinese Journal of Aeronautics [96].

In terms of fundamental process optimization, the precision and consistency of mask electrochemical machining have been significantly enhanced through the coordinated control of mask structure, flow field design, and power parameters. Wang et al. [94] optimized the core mask structure by proposing a conical-hole mask design. It was found that a mask wall angle of 140° effectively reduced flow field vortices and improved electrolyte uniformity, successfully producing high-precision hole arrays with diameter deviation < 0.1 mm and roundness deviation of 12.04 μm. To address the challenge of uneven flow fields in large-area machining, Li et al. [95] designed a serpentine flow channel incorporating internal circular flow guides. This reduced flow velocity fluctuations in the bend regions from ±25% to ±5%, enabling the highly consistent fabrication of a 10 × 20 hole array over a 90 mm × 180 mm area. Furthermore, the optimization of power parameters is crucial. Wang et al. [96] demonstrated that a pulsed power supply (period: 2.5 ms, duty cycle: 20%) effectively suppressed electrolyte bubble accumulation and Joule heating, reducing the conductivity variation from 48.7% under DC power to 6.95%, thereby ensuring the forming accuracy of a 1428-hole array (as shown in Figure 17 and Figure 18).

Regarding novel technological innovations, researchers have overcome the limitations of traditional mask electrochemical machining by introducing multi-field coupling and combined additive-subtractive processes. Yang et al. [97] developed induction electrode through-mask electrochemical micromachining, employing an innovative structure with a “powered electrode and a wireless, inductively coupled workpiece”. This configuration achieved self-termination, gradient etching, and parallel processing capabilities, reducing non-uniformity from 50% to 3.8% and enabling the efficient fabrication of microstructures with gradient depths. On the other hand, to address surface defects in mask electrodeposition, Zhang et al. [98] proposed a mask-based electrochemical hybrid additive-subtractive manufacturing method. Through a “deposit-first, dissolve-later” process that exploits the electric field line concentration effect on protrusions, the height difference on the surface of micro-pillars was reduced from 13 μm to 2 μm, significantly improving surface flatness and providing a new method for high-precision micro-pillar array fabrication.

In the shaping of specific microstructures, mask electrochemical machining has demonstrated a strong capability for manufacturing complex microstructures through customized processes and mask designs. Chen et al. [99] machined micro-grooves using a porous cathode integrated with a mask and a jet-induced flow field, which effectively enhanced the mass transport process. The micro-groove depth deviation was reduced to ±4.9 μm, and a width uniformity of 96% was achieved. For more complex semi-cylindrical micro-grooves and dense arrays, Miao et al. [100] utilized elliptical-hole masks combined with a scanning motion. By designing the elliptical holes in a staggered arrangement, micro-groove arrays with vertical sidewalls were successfully fabricated at a minimal pitch of 160 μm. Furthermore, in the machining of micro-slit arrays, Chen et al. [101] established a numerical model that revealed the dynamic evolution of the electric field during the process. Based on this, process parameters were optimized, ultimately producing high-quality micro-slit arrays with controllable taper and burr-free edges on 50 μm thick stainless steel.

In summary, through electric field confinement by the mask and the synergistic optimization of multiple parameters, mask electrochemical machining enables “arrayed, high-precision, low-damage” micro-fabrication. Via multi-dimensional optimization of fundamental processes and the innovation of new technologies, this technique has become one of the key methods for shaping arrayed microstructures, demonstrating broad application prospects, particularly in fields such as aerospace, microelectronics, and energy devices.

#### 2.3.6. Jet Electrochemical Machining

**Figure 19:**
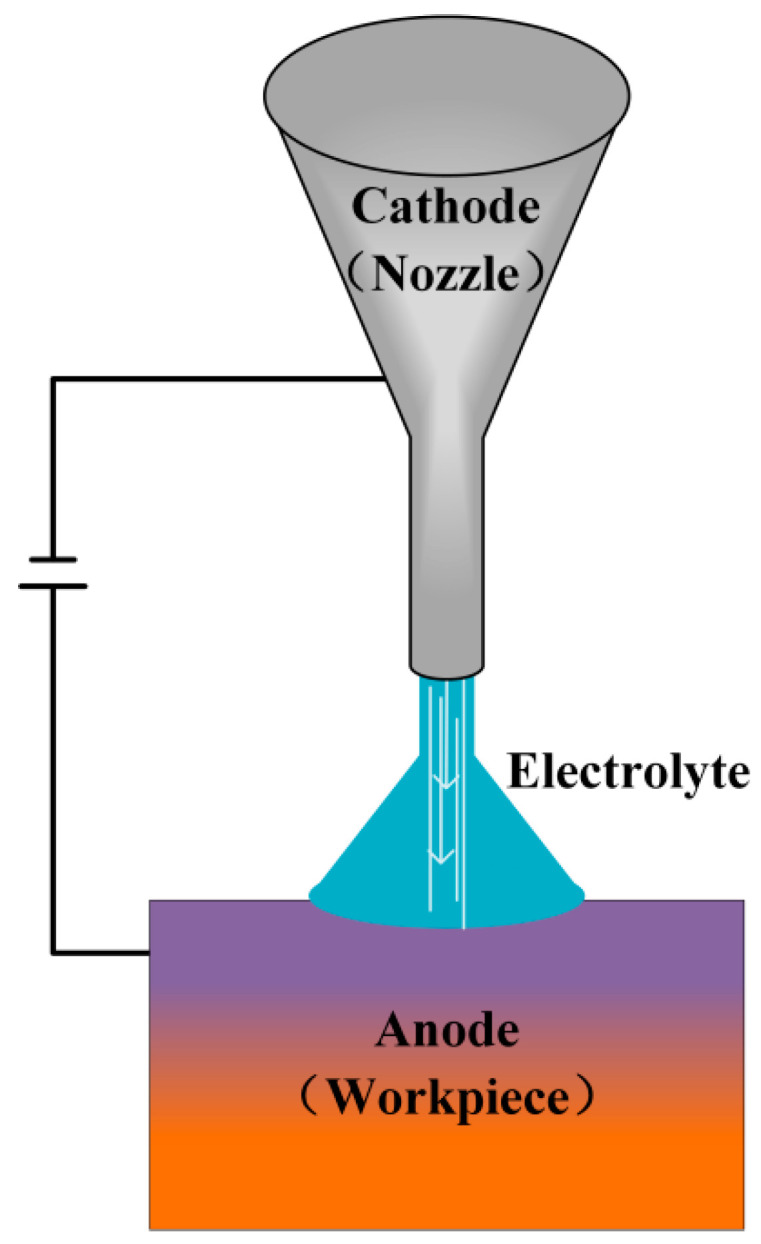
Schematic diagram of jet electrochemical machining principle.

Jet electrochemical machining utilizes high-pressure electrolyte jets to achieve localized anodic dissolution, characterized by non-contact processing, absence of heat-affected zones, and compatibility with difficult-to-machine materials. This technology enables efficient fabrication of microstructures such as microchannels and multi-grooves, establishing it as a key technique in micro-material forming. The core principle involves using a metal nozzle as the cathode and the workpiece as the anode. A high-pressure electrolyte is ejected through the nozzle to form a high-speed jet, inducing anodic dissolution exclusively in the contact area (as shown in Figure 19). Diverse structures can be formed through trajectory control without the need for complex customized cathodes. However, conventional processes face limitations such as constraints in micro-scale forming and stray corrosion [102].

Advances in jet electrochemical machining for microstructure forming are primarily reflected in process innovation, material compatibility, and theoretical modeling. To enhance multi-channel processing capability, Chen et al. [103] implemented a flexible insulating mask integrated with micro-through-holes at the nozzle tip, enabling the formation of microchannel arrays with high length-to-width ratios using a single nozzle. This process, involving two-step machining with a 90° workpiece rotation, produced cross-microchannels where the intersection depth reached 35 μm due to secondary machining. For difficult-to-machine materials like TB6 titanium alloy, Liu et al. [104] demonstrated that NaCl electrolyte effectively penetrates the surface oxide layer to achieve stable dissolution, achieving high material removal rates and low surface roughness under specific parameters. To further improve processing efficiency, Malik et al. [105] developed a laser-assisted jet electrochemical machining technique. This approach leverages thermo-electrochemical synergistic effects, significantly enhancing the material removal rate for Inconel-718 while improving hole taper and surface quality. Theoretically, Guo et al. [106] established a multi-scale 3D finite element method model that successfully predicts the morphological evolution of microchannels, with absolute errors on the order of several micrometers (e.g., approximately 6 to 8 μm for different workpiece materials) between predictions and experimental measurements. This model, validated for both micro-scale (nozzle diameter 130 μm) and meso-scale (nozzle diameter 1 mm) jet electrochemical machining processes, revealed the influence of nozzle configurations on current density distribution and machining outcomes. For engineering applications, Luo et al. [107] introduced a row-based tubular electrode technology, where insulating coatings effectively suppress stray corrosion. After optimizing tube spacing, high-precision groove arrays were fabricated over large workpiece areas.

In summary, jet electrochemical machining has achieved comprehensive improvements in micro-forming precision, material adaptability, and engineering applicability through process refinements, hybrid enhancements, theoretical modeling, and equipment optimizations. Future efforts should focus on enhancing control at micro-nano scales and expanding large-scale applications in high-end manufacturing, facilitating the transition of this technology from laboratory research to industrial practice.

The aforementioned six microscopic ECM technologies, including Wire Electrochemical Turning and Electrochemical Micro-Milling, have effectively broken through the bottlenecks of traditional micromachining in material adaptability, precision control, and structural complexity from different dimensions, and their applications have been achieved in key fields such as aerospace and microelectronics. Currently, issues such as the uniformity of field distribution at the micro-nano scale, multi-energy field integration, and the development of green electrolytes remain to be addressed. Future efforts need to be directed towards promoting the transition of microscopic ECM from laboratory-level precision to industrial-scale stability, so as to provide core technical support for the miniaturization of high-end devices.

### 2.4. Summary of Typical ECM Technologies

To systematically compare the differences in core characteristics, performance, and application scope of the aforementioned ECM technologies (addressed in Section 2.1, Section 2.2 and Section 2.3), Table 1 summarizes 12 typical ECM processes in terms of core principles, machining performance, applicable materials, typical applications, and technical limitations, providing a reference for technology selection in practical manufacturing.

## 3. Typical Applications and Case Analysis

Section 2 systematically elaborates on the principles, research progress, and technical characteristics of ECM in surface quality enhancement, macro-scale material shaping, and micro-scale material shaping—verifying its feasibility for difficult-to-machine materials such as Ti-6Al-4V and 4H-SiC. To further demonstrate the engineering value of these technologies, Section 3 focuses on typical application scenarios (medical devices, energy equipment, aerospace components) and analyzes the performance of ECM in practical manufacturing through specific cases and quantitative metrics—bridging the gap between theoretical research and industrial application.

### 3.1. Typical Applications of Surface Quality Improvement ECM

#### 3.1.1. Medical Devices

Electrochemical processing has enabled significant advancements in medical device manufacturing, particularly for implant surface treatments that enhance biocompatibility through precise control of surface characteristics. These treatments are primarily implemented via electrochemical reactions such as anodic oxidation and electrochemical etching, which allow controlled modification of surface topography and chemistry to optimize biological responses. Electrochemical polishing technology aimed at enhancing surface integrity improves the biocompatibility of implants by eliminating surface irregularities and contaminants. Wang et al. [108] used electrochemical anodizing technology to modify the surface of metal implants with nanotubes, successfully improving the biocompatibility and bone integration ability of the implants. Gao et al. [109] used electrochemical methods such as low-voltage anodizing, micro arc oxidation, and electrodeposition to perform surface engineering treatment on titanium based alloys, enhancing their biological activity and antibacterial properties. Al-Hashedi et al. [110] combined electrochemical treatment with mechanical brushing to disinfect contaminated titanium surfaces, effectively removing bacteria while preserving surface integrity. These technological breakthroughs enhance not only the biological functionality and safety of implants but also provide essential manufacturing support for developing minimally invasive surgical tools and fabricating precision drug delivery systems. This advancement thereby drives innovation in surface processing technology for medical applications.

#### 3.1.2. Energy Industry

Electrochemical processing plays a key role in solar cell manufacturing by enabling precise surface morphology control, multi-material compatibility, and environmentally friendly characteristics [111]. Researchers such as Lee et al. [8,112] employed electrochemical mechanical polishing to treat stainless steel substrates. Through optimization of electrolyte composition, abrasive particle size, and process parameters, they reduced the surface roughness of SS304 and SS430 substrates from 35 nm to 10 nm. X-ray photoelectron spectroscopy and secondary ion mass spectrometry analyses revealed that a passivation layer (Cr_2_O_3_/Fe_2_O_3_) formed on the SS304 and SS430 substrates during electrochemical mechanical polishing effectively suppressed Fe/Cr impurity diffusion, ultimately increasing the conversion efficiency of amorphous silicon solar cells to 5.1–5.4%. This technological breakthrough not only enhances the photoelectric performance of photovoltaic materials, but also provides an environmentally friendly and high-precision processing solution for the large-scale preparation of efficient solar cells.

#### 3.1.3. Technological Significance of Surface Quality Improvement ECM

ECM technologies have driven significant advancements in medical and energy sectors through surface engineering innovations. Medical applications focus on nanoscale surface modifications: (1) the TiO_2_ nanotube arrays with diameters ranging from 15 to 800 nm prepared by anodic oxidation enhance biocompatibility and osseointegration; (2) improved bioactivity and antibacterial properties of titanium alloys through micro-arc oxidation and electrodeposition, as shown in Table 2. In the energy sector, electrochemical mechanical polishing optimizes solar cell substrates by suppressing impurity diffusion, achieving surface roughness Ra below 0.35 µm through electrolyte and voltage optimization. Future development requires reliability verification for clinical implants and environmental impact assessments of electrochemical processes.

### 3.2. Typical Applications of ECM in Macroscopic Material Forming

#### 3.2.1. Aerospace Field: Turbine Blades

In aerospace applications, the critical role of electrochemical surface treatment in manufacturing high-temperature components has been systematically highlighted by recent research. ECM, with supplementary mechanical processes employed as necessary, has emerged as a pivotal solution for addressing challenges in processing difficult-to-machine aerospace materials. Thermal and mechanical limitations inherent to conventional methods are effectively overcome through the leverage of electrochemical dissolution, as consistently demonstrated in the precision machining of titanium- and nickel-based alloys.

ECM has irreplaceable technological advantages in the manufacturing of aerospace turbine blades. It successfully solves the complex surface machining problems of difficult to machine materials such as titanium/nickel based alloys through its non thermal damage and non-contact material removal characteristics. Research has shown that by combining multi-physics field simulation with cathode design innovation, ECM technology can achieve a blade profile accuracy of 0.1 mm and a surface roughness (Ra) of 0.8 µm, with excellent surface integrity, significantly better than traditional machining methods [113]. Further optimization of electrolyte flow using vertical flow mode reduces bubble generation rate by 2.4% and temperature rise by 0.6 K, improves conductivity by 0.47 S/m, achieves machining deviations of 3.4~75.6 µm and surface roughness Ra below 0.35 µm [114]. In experiments with specific electrolyte compositions, this technology can break through the bottleneck of dissolution control for difficult-to-machine materials and has been applied to the precision machining of new high-temperature alloys such as γ-TiAl by optimizing the electrolyte flow field and pulse parameters, providing key manufacturing support for the lightweight and long-life design of high-temperature components in aircraft engines [115].

#### 3.2.2. Aerospace Field: Thin Walled Receiver

**Figure 20:**
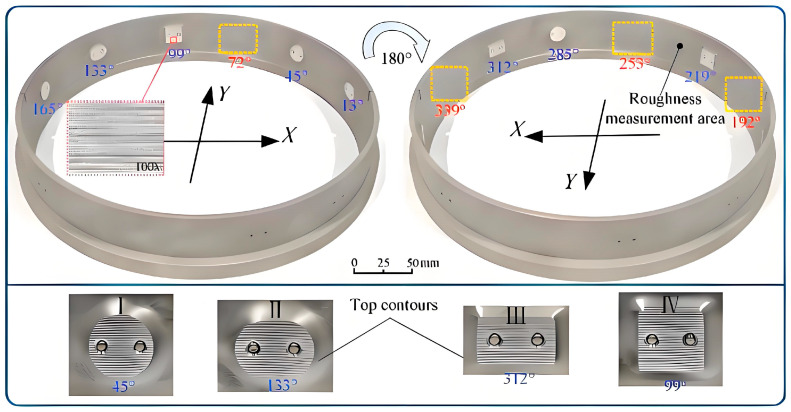
Anode workpiece machined with a flexible cathode tool. Reprinted from Precision Engineering, Copyright (2024), with permission from Elsevier [118].

ECM has shown significant advantages in the manufacturing of aerospace thin-walled casings, with its non-contact ion dissolution characteristics effectively solving problems such as deformation and stray corrosion in traditional machining. Cao et al. [116] successfully reduced the sidewall taper of a titanium alloy thin-walled casing convex structure from 25.5° to 1.11° using counter-rotating electrochemical machining integrated with insulation coating technology. This approach achieved a surface roughness Ra of 1.8 µm and overcame the critical challenge of stray current control. Zhu et al. [117] further proposed the pure rolling cathode ECM method, established a multi physics field coupling model, and achieved a thickness error of 0.12 mm and a MRR of 1.01 cm^3^/min in the processing of nickel based alloy thin-walled plates. Zhou et al. [118] proposed a co-rotating electrochemical machining method, which successfully achieved efficient and precise machining of non array distributed convex structures on the inner surface of annular parts by designing flexible cathode tools and establishing a multi physics field coupling model. The experimental results demonstrate successful machining of a 6 mm-high convex structure with 1.2 mm sidewall thickness on nickel-based alloy. This method achieved a surface roughness Ra of 0.494 µm while overcoming the technical bottleneck of complex structure positioning in traditional processing. As illustrated in Figure 20, a flexible cathode tool processes the anode workpiece during this operation. These technological innovations not only improve processing efficiency and accuracy, but also provide key support for lightweight design of aircraft engines, meet the high-precision manufacturing needs of complex structures and difficult to machine materials, and promote the innovation of thin-walled component manufacturing technology in the aerospace field.

#### 3.2.3. Technological Significance of ECM in Macroscopic Material Forming

ECM has emerged as a critical aerospace manufacturing technology owing to its non-thermal characteristics and non-contact ionic dissolution mechanism. This approach effectively addresses dissolution control challenges for difficult-to-machine materials while enabling complex structural fabrication. Documented applications confirm enhanced manufacturing precision and structural reliability for aeroengine components and thin-walled systems, thereby supporting lightweight design and extended service life requirements, as shown in Table 3.

### 3.3. Typical Applications of ECM in Microscopic Material Forming

#### 3.3.1. Micro Electrode Fabrication for Precision Machining

ECM has enabled breakthroughs in microelectrode fabrication, addressing critical challenges in micro-nano scale manufacturing. Saha et al. [119] employed electrochemical etching to fabricate tungsten carbide microelectrodes with diameters as small as 3.33 μm for micro-EDM (Electrical Discharge Machining) applications. Process optimization via Taguchi methodology revealed that alternating current supply voltage and spindle rotation speed significantly govern electrode dimensional accuracy. Field emission scanning electron microscopy analysis confirmed conical electrode profiles, while EDX results indicated negligible material degradation during etching. This technique overcomes microtool batch production limitations, as ECE insulating masks suppresses stray corrosion and ensures uniform material removal. The fabricated electrodes were successfully implemented in micro-EDM of Ti-6Al-4V, achieving crater diameters of 23.18 μm with submicron precision, demonstrating critical role of ECM in bridging microtool fabrication and high-precision machining.

#### 3.3.2. Microfluidic Device Fabrication on Printed Circuit Boards

ECM has emerged as a pivotal technology for integrating microfluidic structures with electronic substrates. Singh and Bhattacharya [120] implemented electrochemical micromachining on FR4 printed circuit boards to fabricate micromixers with feature sizes down to 243 μm. Using a single-point tool with a 150 μm tip diameter, the researchers achieved surface roughness between 3.0459 and 7.2404 μm with ±0.025 μm tolerance via the process, surpassing traditional micro-milling in resolution and environmental compatibility. COMSOL simulations optimized mixer geometry for laminar fluid mixing, while epifluorescence microscopy validated efficient fluid blending at Reynolds numbers of 48.5–146.37. This approach eliminates lithography-dependent masking, enabling rapid prototyping of PCB-integrated microfluidic systems for lab-on-a-chip applications.

#### 3.3.3. Micro-Channel Array Fabrication for Fuel Cell Bipolar Plates

In energy device manufacturing, ECM has revolutionized the fabrication of micro-channel arrays in fuel cell bipolar plates. Chen et al. [121] proposed electrochemical direct writing processing to generate 10 parallel microchannels each 60 mm long on a 0.5 mm thick stainless steel plate. The technique employs an insulated mask with micro-through-holes integrated into a metallic nozzle, enabling single-step patterning under pulse parameters of 20 V, 20% duty cycle, and 2 kHz frequency. Confocal microscopy measurements revealed channel dimensions of 302 ± 3.53 μm width and 95.9 ± 1.34 μm depth, demonstrating minimal stray corrosion. The flow field simulation revealed that electrolyte accumulation at channel endpoints influenced profile consistency, while optimized pulse parameters mitigated this effect. This method addresses mass production needs for proton exchange membrane fuel cells, achieving high-dimensional uniformity in micro-channel arrays.

#### 3.3.4. Technological Significance of ECM in Microscopic Material Forming

The applications in microelectrode fabrication, microfluidic integration, and fuel cell component manufacturing collectively demonstrate the versatility of ECM in micro/nano fabrication. By combining electrochemical dissolution with precision motion control, these methods enable feature sizes down to submicron scales while maintaining material integrity, as shown in Table 4. The use of pulse processing and insulated masks in ECM suppresses electrolytic product accumulation and stray corrosion, addressing long-standing challenges in microscale manufacturing. These advancements not only support the miniaturization of medical devices and energy systems but also establish ECM as a cornerstone technology for next-generation micro/nano manufacturing.

## 4. Challenges and Development Directions

### 4.1. Existing Technological Bottlenecks

Significant technological bottlenecks persist in ECM, primarily concerning: (1) dynamic gap control instability, (2) environmental sustainability challenges, and (3) abrasive tool degradation. These limitations collectively impede the technology’s progression towards high-precision manufacturing and large-scale industrial adoption.

#### 4.1.1. Dynamic Gap Control Challenges

The instability of the dynamic inter-electrode gap (IEG) in ECM is not simply a matter of accuracy, but rather a complex consequence arising from the coupling of multiple physical fields (electrical, flow, and thermal) during the process, a point which was not sufficiently elaborated in the original text. Research findings have clearly established that non-uniform electrolyte flow and localized distortions of the electric field are the primary causes of IEG fluctuations. For instance, in the electrochemical milling of deep-narrow grooves, it was observed by Chen et al. [57] that the use of a conventional flat-end tube electrode resulted in a 41.7% difference in electrolyte velocity between the groove bottom and the sidewall (decreasing from 0.24 m/s to 0.14 m/s). This flow field heterogeneity led to a 35% variation in local current density, directly causing the IEG to fluctuate between 80 μm and 150 μm, and ultimately resulting in a groove width error of up to 0.32 mm.

Furthermore, the accumulation of electrochemical reaction products (e.g., hydrogen bubbles, metal hydroxides) further exacerbates IEG instability. In experiments involving rotating cathode electrochemical milling, the adherence of hydrogen bubbles generated at the cathode to the electrode surface was observed by Shen et al. [58], forming a “gas film” with a thickness of 5–10 μm. This film reduces the effective conductive area of the electrode, causing a 22–28% decrease in current density and inducing periodic IEG oscillations with a frequency of 0.5–1 Hz. Even with the implementation of intelligent control strategies such as the linear time-varying model predictive control proposed by Boxhammer et al. [27], a response lag of 15–20 ms persists when addressing sudden IEG changes (e.g., those induced by electrolyte temperature increases). Consequently, the precision requirements for optical components (e.g., dimensional tolerances of ±5 μm), as stipulated in relevant research, are difficult to meet.

These findings confirm that the challenge of dynamic gap control is fundamentally a problem of coordinated multi-physics field regulation, rather than an issue of single-parameter control. The original text failed to integrate these literature findings, resulting in an overly generalized description of the challenge.

#### 4.1.2. Environmental Limitations

Traditional aqueous electrolytes, such as NaNO_3_, are widely employed in electrochemical machining processes, including those for Inconel 718 [24]. However, the use of these electrolytes poses significant environmental burdens, primarily associated with post-treatment requirements. For instance, in the pulsed electrochemical machining of Inconel 718 using NaNO_3_ electrolyte, subsequent treatment by neutralization and precipitation is required to address environmental concerns, since discharge of untreated electrolyte may lead to contamination issues.

In comparison, improved environmental compatibility is offered by green electrolytes represented by solid-state systems, through the complete elimination of liquid wastewater generation. A Nafion/CeO_2_ composite solid electrolyte was developed by Murata et al. [35] for 4H-SiC polishing, which entirely avoids both liquid electrolyte handling and wastewater discharge. However, the large-scale industrial application of this solid electrolyte system is hindered by its substantially higher material and preparation costs relative to conventional aqueous electrolytes. Further optimization of green electrolyte design was demonstrated by Inada et al. [36], where polystyrene sulfonic acid was utilized as a matrix for fabricating solid electrolyte composite liners. Efficient polishing of 4H-SiC wafers was achieved with this configuration, obtaining a material removal rate of 14.3 μm/h while maintaining environmental compatibility. Despite these advancements, the commercial viability of PSS-based solid electrolytes remains constrained by cost considerations in high-volume manufacturing applications.

This fundamental contradiction, wherein traditional aqueous electrolytes exhibit lower initial implementation barriers but necessitate expensive environmental post-treatment, while green solid electrolytes minimize environmental treatment requirements but encounter economic scalability limitations, represents a crucial environmental bottleneck that was not sufficiently examined in the original draft.

#### 4.1.3. Abrasive Tool Degradation

Tool degradation in electrochemical processes involves complex interactions between electrochemical corrosion and mechanical wear, a phenomenon that has been systematically investigated in recent studies.

Tool structural weakening is primarily induced through two corrosion pathways: selective matrix dissolution and interfacial corrosion at abrasive-bonding junctions. During electrochemical grinding of GH4169 alloy, anodic dissolution of the Cu-based bonding agent in 10 wt% NaCl electrolyte was documented by Xue et al. [37], generating 1–3 μm diameter micro-pits that reduced effective abrasive bonding area by 25–30%. This degradation leads to abrasive detachment under 50~80 N grinding forces, with dislodged particles acting as third-body abrasives to increase wear rate by 40–50%.

Additionally, low-hardness (HV 50~80) corrosion products like Cu(OH)_2_ provide minimal mechanical resistance. In Ti-6Al-4V grinding, periodic removal of the Cu(OH)_2_ film under cyclic stress was reported by Wang et al. [43], establishing a continuous “corrosion-peeling-recorrosion” cycle that reduces tool life to 60–70% of pure mechanical grinding.

Wear behavior varies significantly with tool design. Cu-based conductive wheels [37] exhibit 8–12% tool wear rate (TWR) due to non-passivating matrix dissolution. Insulated tools [39] achieve 1–3% TWR through complete electrochemical isolation by epoxy resin coatings. Robotic ECMP cathodes [34] demonstrate minimal 0.5–1% TWR via protective TiO_2_ passivation films.

These systematic wear variations, determined by material corrosion resistance and structural design, constitute a critical aspect omitted from the original discussion.

### 4.2. Frontier Research Directions

#### 4.2.1. Green Processing Technology

Green machining technology has emerged as an important direction in ECM, owing to its breakthrough in balancing sustainability and machining performance. Conventional ECM has relied on strongly acidic electrolytes, which can achieve precision machining but are accompanied by high pollution risks, excessive energy consumption, and equipment corrosion problems. Recent studies have significantly reduced toxic gas emissions and nanoparticle generation through the development of eco-friendly electrolyte systems while maintaining material dissolution efficiency, thereby meeting environmental regulations such as restriction of hazardous substances requirements. Concurrently, process parameter optimization has been proven to effectively inhibit harmful byproduct formation, demonstrating universal applicability to various manufacturing processes including additive manufacturing, rolling, and milling. These advancements not only resolve the environmental deficiencies inherent in traditional ECM but also drive the technology toward higher efficiency, lower consumption, and enhanced safety, consequently establishing this field as a current research hotspot. For example, Dadi et al. [122] developed an electrochemical polishing process utilizing eco-friendly electrolytes and nano-polishing tools for 17-4PH stainless steel additive manufacturing components. This method reduced surface roughness values from 0.8 to 1.2 µm to 0.04 to 0.05 µm, demonstrating its efficacy on surfaces produced by diverse manufacturing techniques. Singh et al. [123] analyzed smoke composition from different electrolytes in electrochemical discharge machining via fourier transform infrared spectroscopy and FE-SEM. Results showed that NaOH electrolyte at low concentration generated significantly fewer toxic gases and nanoparticles than NaCl and HCl electrolytes. This confirms the feasibility of NaOH as a green electrolyte alternative.

#### 4.2.2. Intelligent Control Strategy

Intelligent control technology has been established as a frontier direction in ECM/EDM (Electrical Discharge Machining), stemming from its pronounced advantages in multi-physical field coupling regulation and process coordination optimization. Conventional ECM/EDM processes have been constrained by bottlenecks including low machining efficiency, challenging parameter matching, and unstable dimensional control. Intelligent control collects electrical pulse signals such as voltage, current and spark frequency in real time, and combines machine learning algorithms to build cross-process prediction models to achieve dynamic compensation in the processing process. For example, Chuang et al. [124] established a neural network model to predict machining dimensions by analyzing the electrical signal characteristics of ECM and EDM. They reduced the machining time of Inconel 718 alloy by 80% and achieved a prediction accuracy of 0.01 mm^2^. This technology effectively solves the contradiction between MRR and surface quality in traditional processes through feature extraction and pattern recognition, while reducing reliance on empirical parameters. In addition, intelligent control technology can also be combined with multi physics field simulation to optimize electrolyte flow and electrode feed strategies, achieving precise control of the machining process and providing efficient solutions for complex surface machining and new material applications. Therefore, it has become the core direction of current research.

#### 4.2.3. Cross Scale Processing

Cross-scale machining technology has been established as a frontier direction in ECM, originating from its unique advantages in coordinated manufacturing of multi-scale structures. Conventional ECM processes have been constrained by their single-scale processing capability, having been challenged in simultaneously ensuring macro-scale structural accuracy and the integrated manufacturing of micro-scale functional surfaces. Tao et al. [125] achieved cross scale machining of millimeter level cavities and micrometer level superhydrophobic end faces by designing microstructured surface cathodes and combining electric and flow field simulations. The machining accuracy was improved by 38.6% and a superhydrophobic surface with a contact angle of 150.5° was obtained. This technology optimizes the cathode microstructure to regulate the current density distribution, promotes bubble desorption and electrolyte renewal, and breaks through the contradiction between MRR and surface quality in traditional ECM.

#### 4.2.4. Multi Energy Field Combination

**Figure 21:**
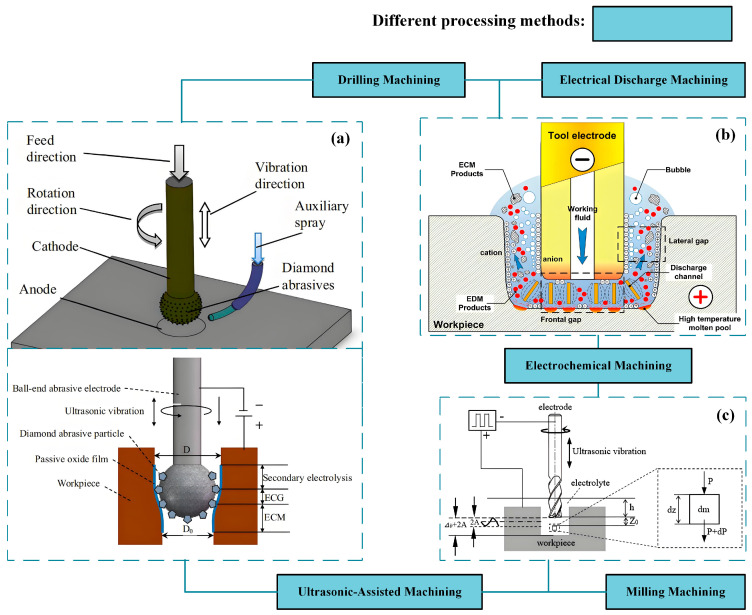
Multi-Energy Field Hybrid Processing Diagram, (**a**) UAECDG. Reprinted from Journal of Advanced Research, Copyright (2020), with permission from Elsevier [126], (**b**) ECDD. Reprinted from CIRP Journal of Manufacturing Science and Technology, Copyright (2020), with permission from Elsevier [127], (**c**) UA-MECM. Reproduced from Sensors [128].

For example, ultrasound-assisted electrochemical drilling and grinding (UAECDG) optimizes the electrolyte flow field through high-frequency vibration, accelerating reaction product discharge and suppressing bubble aggregation. This achieves machining accuracy of 1.1 ± 0.01 mm for 304 stainless steel small holes with surface roughness Ra at 0.31 µm [126]. Electrochemical discharge drilling (ECDD) leverages discharge-dissolution duality in low-conductivity salt solutions, producing recast-layer-free microholes on nickel-based alloys exhibiting surface roughness Ra of 1.69 µm, significantly enhancing surface integrity [127]. Rotating ultrasonic vibration-assisted micro electrochemical milling (UA-MECM) technology regulates electric field distribution via vibrational-mechanical interactions, reducing surface roughness from an initial Ra of 0.83 µm to a final Ra of 0.26 µm while fabricating complex 3D structures [128]. These technologies resolve the efficiency-surface quality conflict through complementary energy-field interactions, as shown in Figure 21.

## 5. Conclusions and Prospect

ECM is established as a non-traditional material removal method based on controlled anodic dissolution, distinguished by its absence of tool-workpiece contact and material hardness independence. This technology effectively addresses machining challenges presented by advanced materials including titanium alloys, nickel-based superalloys, and hard-brittle semiconductors in high-end manufacturing applications. Its implementation provides essential support for fabricating lightweight complex aerospace components and enabling high-density micro-nano device integration. Specific applications demonstrate its critical role in aerospace engineering through solving deformation and stray corrosion issues in turbine blades and thin-walled casings, while in semiconductor manufacturing it enables high-precision surface finishing and micro-feature fabrication through derived processes like electrochemical mechanical polishing.

The broader implementation of ECM in high-end manufacturing is nevertheless hindered by several persistent technical constraints. Processing precision is compromised by dynamic inter-electrode gap instability resulting from multi-physical field coupling effects, including non-uniform electrolyte flow and reaction product accumulation. Significant environmental and economic trade-offs are observed between conventional aqueous electrolytes requiring extensive post-treatment and green solid electrolytes limited by scalability constraints. Furthermore, process stability in long-cycle micro-nano machining is undermined by abrasive tool degradation through synergistic electrochemical-mechanical mechanisms, consequently restricting reliability in high-density device fabrication.

Future development trajectories for ECM are directed toward resolving these fundamental limitations through targeted research initiatives. Green processing technologies incorporating eco-friendly electrolyte systems are anticipated to reduce environmental impact while maintaining machining performance for scalable lightweight component production. Enhanced process stability is expected through intelligent control systems implementing multi-physical field modeling and real-time parameter optimization. Additionally, cross-scale manufacturing capabilities are projected to be advanced through multi-energy field hybridization approaches including ultrasonic and laser assistance, effectively bridging macro-structural fabrication with micro-nano functional integration. These coordinated advancements will solidify ECM as an enabling manufacturing technology, driving innovation across the aerospace, semiconductor, and energy sectors while supporting the comprehensive upgrade of high-end manufacturing capabilities.

## Figures and Tables

**Table 1:** Comparative Table of 12 Electrochemical Machining (ECM) Technologies.

No.	Technology Name	Machining Performance	Applicable Materials	Key Technical Limitations	**Typical Applications**
1	Pulsed Electrochemical Polishing	Surface Roughness: Low (Ra 0.06–0.3 μm for Inconel 718/Nimonic-263) MRR: Medium (10–20% higher than DC electrochemical polishing)	Nickel-based superalloys (Inconel 718, Nimonic-263), Invar sheets, stainless steels (304)	Dynamic IEG control lags 15–20 ms for sudden disturbances Electrolyte machinability depends on passivation capacity	Precision molds, aerospace turbine blade sealing surfaces, high-gloss metal parts
2	Electrochemical Mechanical Polishing	Surface Roughness: Low (Ra 0.449 nm for 4H-SiC, Sa 0.5–0.66 nm for Ti-6Al-4V) MRR: Slow (2.3 μm/h for 4H-SiC, 147.02 nm/min for cobalt)	Third-generation semiconductors (4H-SiC, GaN), titanium alloys (pure Ti, Ti-6Al-4V), stainless steels (304, 316L), tantalum, AM components (17-4PH, Ti-6Al-4V AM parts)	Difficult to control electric field distribution uniformity High cost of solid electrolytes (e.g., Nafion/CeO_2_ pad)	Semiconductor substrates (4H-SiC wafers), titanium medical implants, precision optical components
3	Electrochemical Grinding	Surface Roughness: Medium (Ra 0.35–1.8 μm for GH4169/Ti-6Al-4V) MRR: Fast (10–30 mm^3^/min for GH4169/Inconel 718)	Difficult-to-machine alloys (GH4169, Ti-6Al-4V, Inconel 718), cemented carbides, ceramics, quenched steels (42CrMo4)	Abrasive tool degradation (Cu-based binder corrosion, 8–12% wear rate) Non-uniform electrolyte flow causes local overcutting	Aerospace turbine blades, precision gears, silicon anodes for batteries
4	Electrochemical Milling-Grinding	Surface Roughness: Medium (Ra 1.06 μm for Ti-6Al-4V, Ra 0.37 μm for titanium matrix composites) MRR: Fast (216.6 mm^3^/min for Inconel 718, 248.3 mm^3^/min for Ti-6Al-4V)	Nickel-based alloys (Inconel 718), titanium alloys (Ti-6Al-4V), SiCP/Al composites, (TiB+TiC)/Ti6Al4V composites	Uneven electrolyte distribution in deep grooves Reinforced phases (SiC/TiB) cause local tool wear	Aerospace complex 3D grooves, thin-walled components, titanium matrix composite parts
5	Electrochemical Milling	Surface Roughness: Medium–Low (Ra 0.24–0.75 μm for Ti-6Al-4V/316L, Ra 0.06–0.08 μm for Nimonic-263) MRR: Fast (20–282.9 mg/min for Ti-6Al-4V with ultra-high current density)	Titanium alloys (Ti-6Al-4V, TB6), nickel-based superalloys (GH4169, Inconel 718, Nimonic-263), stainless steels (316L, 304)	Unstable dynamic IEG (80–150 μm fluctuation) Edge stray corrosion (needs insulated cathode)	Aerospace thin-walled casings, deep-narrow grooves, TB6 titanium alloy flat surfaces
6	Electrochemical Turning	Surface Roughness: Medium–Low (Ra 0.222–2.414 μm for Ti matrix composites/TB6, Ra 0.315 μm for Ni-based alloys) MRR: Medium–Fast (167.1% higher single-cycle removal for Ti composites)	Titanium alloys (Ti-6Al-4V, TB6), nickel-based cast superalloys, titanium matrix composites	Shaft shoulder overcutting (needs sandwich cathode) Cannot machine non-rotationally symmetric structures	Aerospace engine shafts, cylindrical TB6 components, large-allowance revolving parts
7	Wire Electrochemical Turning	Surface Roughness: Medium–Low (micron-scale precision for tungsten micro-rods) MRR: Slow (micro-scale parts, no high MRR data)	Tungsten, difficult-to-machine micro-scale metals	Difficult to control wire tension (affects precision) High requirement for bipolar pulse matching	Tungsten micro-rods (φ11 μm, aspect ratio 36), micro-scale revolving parts
8	Electrochemical Micro-Milling	Surface Roughness: Low (Ra 0.1 μm for Haynes-188, Ra 0.125 μm for nickel-based alloys) MRR: Slow (aspect ratio 6.1 blind grooves)	Nickel-based alloys (GH3030, Haynes-188), titanium alloys, cemented carbides	Hard to prepare micro-electrodes (needs in-situ 10 μm cylindrical electrodes) Slow electrolyte renewal in microcavities	MEMS 2D shapes (25 μm-wide), 3D stepped structures (45 μm-deep), high-aspect-ratio microgrooves
9	Electrochemical Micro-Turning	Surface Roughness: Low (Ra 0.08–0.15 μm for 1.4301 stainless steel, Ra 0.15–0.5 μm for micro-shafts) MRR: Slow (5–65% cutting force reduction)	Stainless steels (1.4301), titanium alloys (Ti-6Al-4V), micro-scale difficult-to-machine metals	Poor synergy between workpiece rotation and pulse parameters Micro-shaft shoulder overcutting	Micro-shafts (20% less shoulder overcut), medical micro-devices
10	Wire Electrochemical Cutting	Surface Roughness: Low (Ra 1.01 μm for 304 stainless steel, Ra 0.86 μm for Inconel-718, Ra 0.108 μm for NiTi) MRR: Medium–Slow (1.7–5.0 μm/s multi-wire feed)	Titanium alloys (Ti-6Al-4V, γ-TiAl), NiTi shape memory alloys, pure tungsten, stainless steels (304, 1.4301)	Poor multi-wire processing consistency (±1.5 μm slit width deviation) Needs precise wire vibration control (2.5–15 mm amplitude)	Fuel cell micro-slit arrays, pure tungsten X-ray gratings, NiTi medical stents
11	Mask Electrochemical Machining	Surface Roughness: Medium–Low (Ra 0.108 μm for NiTi, burr-free stainless steel micro-slits) MRR: Slow (large-area array single-step forming)	Stainless steels (304, 1.4301), titanium alloys (Ti-6Al-4V), monocrystalline silicon, MEMS materials	Uneven large-area flow field (needs serpentine channels) Limited mask service life for batch processing	MEMS arrays, aero-engine cooling hole arrays, fuel cell flow channels
12	Jet Electrochemical Machining	Surface Roughness: Medium (Ra 2.414 μm for TB6 titanium alloy, Ra 0.31 μm for 304 stainless steel) MRR: Medium (14.3 μm/h for 4H-SiC)	Titanium alloys (TB6, Ti-6Al-4V), nickel-based alloys (Inconel-718), stainless steels (304), SiC	Micro-scale forming limitation (nozzle ≥ 130 μm) Stray corrosion in non-jet areas	Microfluidic microchannels, heat sink multi-groove arrays, TB6 curved components

Notes on Performance Grading: Surface Roughness: Low: Ra ≤ 0.5 μm; Medium: 0.5 μm < Ra ≤ 2 μm; High: Ra > 2 μm. MRR: Fast: MRR ≥ 10 mm^3^/min or equivalent; Medium: MRR between “Fast” and “Slow”; Slow: MRR ≤ 5 μm/h or equivalent.

**Table 2:** Key Performance of ECM in Surface Quality Enhancement.

Application	ECM Technology	Key Quantitative Metrics
Ti-6Al-4V Implants	ECMP	Surface roughness (Sa): 0.5–0.66 nm; No subsurface damage
SS304 Solar Substrates	ECMP	Ra reduced from 35 nm to 10 nm; Solar cell efficiency: 5.1–5.4%

**Table 3:** ECM Performance in Aerospace Macro-Component Machining.

Aerospace Component	ECM Technology	Key Quantitative Metrics
Inconel 718 Turbine Blades	Pulsed ECM (Vertical Flow)	Profile accuracy: ±0.1 mm; Ra: ≤0.35 μm; MRR: 25 mm^3^/min
Ti-6Al-4V Thin-Walled Casing	Counter-Rotating ECM	Sidewall taper: 1.11° (vs. 25.5° by traditional ECM); Thickness error: ±0.05 mm

**Table 4:** ECM Performance in Micro-Structure Fabrication.

Micro-Manufacturing Goal	ECM Technology	Key Quantitative Metrics
Tungsten Micro-Electrodes	Electrochemical Etching	Minimum diameter: 3.33 μm; Diameter deviation: ±0.2 μm
SS304 Fuel Cell Micro-Channels	Masked Pulsed ECM	Channel width: 302 ± 3.53 μm; Depth: 95.9 ± 1.34 μm; Sidewall Perpendicularity: 95%

## Data Availability

No new data were created or analyzed in this study.
